# MGSEA – a multivariate Gene set enrichment analysis

**DOI:** 10.1186/s12859-019-2716-6

**Published:** 2019-03-18

**Authors:** Khong-Loon Tiong, Chen-Hsiang Yeang

**Affiliations:** Institute of Statistical Science, Academia Sinica, Taipei, Taiwan

**Keywords:** Gene set enrichment analysis, Multimodal OMIC data

## Abstract

**Background:**

Gene Set Enrichment Analysis (GSEA) is a powerful tool to identify enriched functional categories of informative biomarkers. Canonical GSEA takes one-dimensional feature scores derived from the data of one platform as inputs. Numerous extensions of GSEA handling multimodal OMIC data are proposed, yet none of them explicitly captures combinatorial relations of feature scores from multiple platforms.

**Results:**

We propose multivariate GSEA (MGSEA) to capture combinatorial relations of gene set enrichment among multiple platform features. MGSEA successfully captures designed feature relations from simulated data. By applying it to the scores of delineating breast cancer and glioblastoma multiforme (GBM) subtypes from The Cancer Genome Atlas (TCGA) datasets of CNV, DNA methylation and mRNA expressions, we find that breast cancer and GBM data yield both similar and distinct outcomes. Among the enriched functional categories, subtype-specific biomarkers are dominated by mRNA expression in many functional categories in both cancer types and also by CNV in many functional categories in breast cancer. The enriched functional categories belonging to distinct combinatorial patterns are involved different oncogenic processes: cell proliferation (such as cell cycle control, estrogen responses, *MYC* and *E2F* targets) for mRNA expression in breast cancer, invasion and metastasis (such as cell adhesion and epithelial-mesenchymal transition (EMT)) for CNV in breast cancer, and diverse processes (such as immune and inflammatory responses, cell adhesion, angiogenesis, and EMT) for mRNA expression in GBM. These observations persist in two external datasets (Molecular Taxonomy of Breast Cancer International Consortium (METABRIC) for breast cancer and Repository for Molecular Brain Neoplasia Data (REMBRANDT) for GBM) and are consistent with knowledge of cancer subtypes. We further compare the characteristics of MGSEA with several extensions of GSEA and point out the pros and cons of each method.

**Conclusions:**

We demonstrated the utility of MGSEA by inferring the combinatorial relations of multiple platforms for cancer subtype delineation in three multi-OMIC datasets: TCGA, METABRIC and REMBRANDT. The inferred combinatorial patterns are consistent with the current knowledge and also reveal novel insights about cancer subtypes. MGSEA can be further applied to any genotype-phenotype association problems with multimodal OMIC data.

**Electronic supplementary material:**

The online version of this article (10.1186/s12859-019-2716-6) contains supplementary material, which is available to authorized users.

## Background

Mapping the relation between genotypes and phenotypes is a classical problem in biology. Much of the progress in the post-genomic era lies in the direction of resolving the generalized genotype-phenotype problems. Typically, high-throughput molecular features (genomes, transcriptomes, proteomes, epigenomes, etc.) and physiological traits (cell types, disease risks, prognostic prospects, ethnicity, etc.) of a population of subjects are measured. Scientists aim for identifying a limited number of biomarkers from the molecular features that can predict/categorize the phenotypes. Individual markers are often difficult to interpret and subjected to variations from measurements and targeted cohorts. To alleviate these problems, it is mandatory to combine multiple markers and place them in the context of biological knowledge.

Gene Set Enrichment Analysis (GSEA) [1] is one of the most popular bioinformatics tools toward this end. In the setting where GSEA applies, the “scores” of a large number of genes (typically all protein-coding genes) and a much smaller “gene set” with a known function are provided. The goal is to assess whether the high-scoring genes are enriched with members in the gene set. To achieve this goal, GSEA sorts genes in terms of their scores and establishes a random walk along the sorted genes. It advances one step when hitting a member from the gene set and reverses one step otherwise. The level of enrichment and its statistical significance are quantified by the maximum positive distance from the origin during the random walk. This simple yet powerful method is applicable to a wide range of bioinformatics problems. For instance, one may evaluate the scores of differential expressions between the transcriptomic data of tumor and normal samples and find the enriched functional categories of top-ranking biomarkers.

Despite its strength, GSEA has a major limitation: the score of each gene has to be a scalar. This implies either only one molecular feature is probed or information from multiple features is synthesized into one score prior to the enrichment analysis. When GSEA was first proposed, high-throughput OMIC data were dominated by single-modal measurements such as genome sequencing or DNA microarrays alone. With advance of high-throughput technologies and reduction of their costs, multi-modal OMIC data become increasingly common today. A remarkable example is the Cancer Genome Atlas [2, 3], where the data of 7 molecular features of the same cohort of patients are provided (DNA sequence mutations, mRNA transcripts, microRNA transcripts, CNVs, single nucleotide polymorphisms (SNPs), DNA methylations, protein quantifications and phosphorylations). Numerous methods have been proposed to extend GSEA to multi-platform data (see the literature review below). However, none of them explicitly captures the combinatorial relations of enrichment information from multiple platforms. For instance, differentially expressed and differentially methylated genes between tumors and normal tissues may be both enriched with the cell cycle control pathway. Yet multiple combinatorial relations may yield this enrichment outcome: (1) differentially methylated cell cycle control genes are subsumed to differentially expressed cell cycle control genes, (2) differentially expressed cell cycle control genes are subsumed to differentially methylated cell cycle control genes, (3) differentially expressed and differentially methylated cell cycle control genes are marginally overlapped, (4) differentially expressed and differentially methylated cell cycle control genes are nearly identical. It is not obvious how these combinatorial relations can be distinguished from the canonical GSEA outcomes.

To resolve this problem, we generalize GSEA to multidimensional scores. The method, termed Multivariate Gene Set Enrichment Analysis (MGSEA), constructs similar random walks by counting the union of gene set members from the sorted genes in multiple platform features. Relations between features in gene set enrichment are quantified by comparing the empirical random walks from the joint features and the expected random walks conditioned on subsets of those features. We further derived the combinatorial functions that map multiple features to enrichment outcomes according to the comparison results. To prove the concept, we first demonstrated that MGSEA successfully captured the designed combinatorial relations of gene set enrichment from simulated data. We then applied MGSEA to the multimodal data of TCGA breast cancer and glioblastoma multiforme (GBM). We calculated the mutual information scores of each gene’s mRNA expression, CNV and DNA methylation profiles in delineating known cancer subtypes, and assessed the combinatorial relations of gene set enrichments among the mutual information scores in those three platforms. In breast cancer, the combinatorial patterns dominated by each single platform appeared in comparative numbers of functional categories, while those dominated by mRNA expression moderately surpassed those by CNV and DNA methylation. In GBM, the combinatorial patterns dominated by mRNA expression far exceeded those by the other two platforms. The functional categories belonging to distinct combinatorial patterns were also involved in different oncogenic processes: cell proliferation for mRNA expression in breast cancer, invasion and metastasis for CNV in breast cancer, and diverse processes for mRNA expression in GBM. These findings sustained in two external datasets (METABRIC and REMBRANDT for breast cancer and GBM respectively).

Numerous extensions of GSEA were previously proposed. The SetRank algorithm [4] calibrated the statistical significance of multiple gene sets by considering their overlap and hence reduced false positives. Kim and Volsky [5] developed a modified gene set enrichment analysis method based on a parametric statistical model, which substantially reduced computation time compared to the expensive permutation operations of GSEA. Klebanov *et. al.* treated the expression of each member of the gene set as a random variable and developed a novel test statistic to model the correlations of multiple genes [6]. In the same vein, Clark *et. al.* proposed a dimension reduction method in the expression space spanned by members of a gene set [7]. Those multivariate extensions tackled the dependency between gene sets or members within gene sets but kept unimodal feature scores derived primarily from mRNA expressions.

Several other approaches integrated multi-OMIC data in the gene set enrichment analysis. GeneTrail2 handled data from transcriptomics, proteomics, miRNomics, and genomics but reported the enriched pathways for each platform separately [8]. MONA considered regulatory relations between multimodal measurements (such as inhibitory relations between a microRNA expression and its target mRNA expressions) and applied Bayesian inference to assess gene set enrichment probabilistically [9]. moGSA reported a gene set enrichment score by integrating multi-platform data [10]. Despite the merits of each method, none of them explicitly captures combinatorial relations of feature scores from multiple platforms. A more detailed comparison of MGSEA with these methods is reported below.

## Methods

### Overview of univariate GSEA

We first give a brief summary of univariate GSEA reported in Subramanian et al., [1]. To facilitate calculation of statistical significance we modify the definition of a random walk and make it equivalent to the cumulative distribution function of a random variable. The inputs are a universe gene set *L* with *N* genes and a smaller functional gene set *S* ⊂ *L* with *K* < *N* genes. Each gene in *L* has a scalar feature score (e.g., the t-test score of differential expression between tumor and normal samples). The output is a *p*-value quantifying the statistical significance that top-scoring genes are enriched with members of *S*. The following procedures are executed.

1. Sort genes in *L* according to their scores in a descending order (from the best to the worst ones).
2. Define *x* as the rank of genes in terms of their scores, and *y*(*x*) as the number of genes above/equal to rank *x* that belong to the functional gene set *S*. *y*(*x*) can be viewed as a random walk along the sorted genes. Starting with 0, *y*(*x*) increments by 1 if the gene of rank *x* is a member of *S*, and 0 otherwise.
3. If a feature is informative about *S*, then the top-ranking genes are anticipated to be enriched with members in *S*. Therefore, the random walk would quickly gain a high value and remain stable subsequently.
4. The null hypothesis is that the feature is uninformative about *S*, and thus members of *S* are uniformly distributed in the sorted list. The random walk of the null model thus approximates a straight line ${y}_{\phi }(x)=\frac{K}{N}\bullet x$.
5. The significance of the gene set enrichment is quantified by the positive deviation of the empirical *y*(*x*) from the null model *y*_*ϕ*_(*x*). Specifically, we normalize random walk curves to 0 ≤ *y*(*x*) ≤ 1 and treat them as cumulative distribution functions (CDFs) of random variables. *P*-values are calculated by non-parametric such as the Kolmogorov-Smirnov test, the Mann-Whitney U test, or the permutation test.

**Fig. 1:**
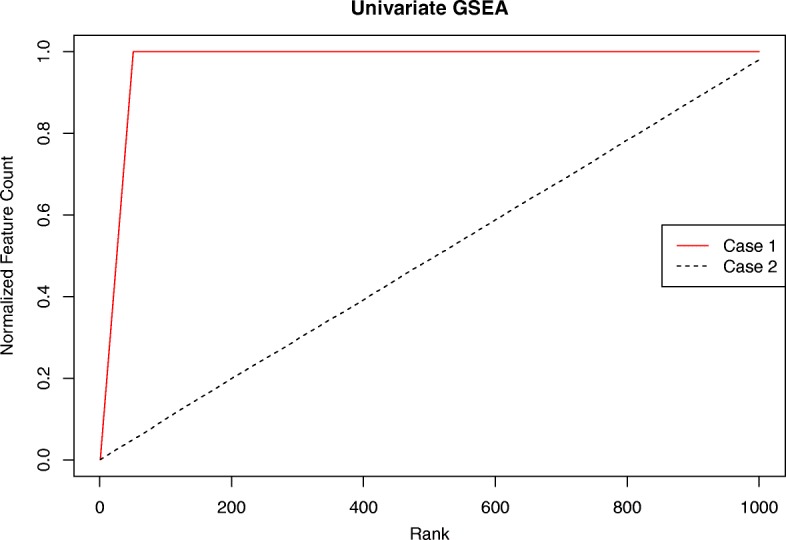
Univariate GSEA random walks of two extreme cases. Case 1: all the gene set members are concentrated at the top 50 genes (solid red line). Case 2: the gene set members are uniformly distributed along all the genes (dashed black line)

A toy example of univariate GSEA is illustrated in Fig. 1. Suppose there are totally 1000 genes (|*L*| = 1000) and 50 of them belong to a functional gene set (| *S* | = 50). In case 1 (solid red line), the gene set members are all concentrated in the top 50 genes. The normalized *y*(*x*) thus linearly ascends from 0 to 1 in a small range (*x* =1–50) and remains at 1 through the remaining ranks. In case 2 (dotted black line), we randomly permute the gene ranks in case 1 10,000 times and plot the mean of the *y*(*x*)^′^*s* from all permutations. The mean random walk resembles a diagonal line connecting (0,0), (1000,1). Cases 1 and 2 represent two extreme conditions where the ranks are either perfectly aligned with or independent of the gene set. Therefore, the random walk of case 1 possesses the maximal positive deviation from the diagonal line, while the mean random walk of case 2 coincides to the diagonal line and has a zero deviation.

### Bivariate GSEA

**Fig. 2:**
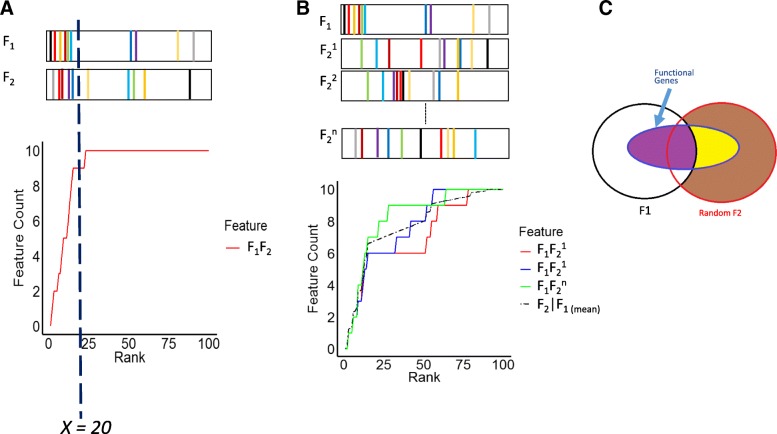
Illustration of bivariate GSEA. Panel A depicts the assessment of *y*_*F*1*F*2_(*x*) at *x* = 20. The two bars on top represent the distributions of functional genes along *F*_1_ and *F*_2_ ranks, where each vertical line with a unique color denotes a distinct functional gene. Here y_*F1F2*_(*20*) =9. Panel B illustrates estimation of *y*_*F*2 ∣ *F*1_(*x*). *F*_1_ is fixed; *F*_*2*_^*1*^*, F*_*2*_^*2*^*, …, F*_*2*_^*n*^ represent distinct random permutations of *F*_2_ ranks. The dashed black line denotes the mean of the random walks over permutations. Panel C elucidates the relations of genes selected from the sorted *F*_1_ and *F*_2_ lists at a given rank *n*. Black and red circles denote the top-*n* genes according to *F*_1_ and randomized *F*_2_ ranks. The blue ellipse denotes the gene set members among the union of genes in the *F*_1_ (black) and *F*_2_ (red) circles. Both *F*_1_ and *F*_2_ circles have the size of *n*. The intersection of the functional genes and *F*_1_ genes (the purple area) has the size of *k*. By taking the union of *F*_1_ and *F*_2_ genes *n*_*extra*_ genes are appended (the brown and yellow areas combined), among them there are *k*_*extra*_ gene set members (the yellow area)

We then consider the simplest extension of GSEA to two features. Two features *F*_1_ and *F*_2_ give rise to two scores for each gene. We sort genes in terms of the two sets of feature scores separately and establish two random walks *y*_*F*1_(*x*) and *y*_*F*2_(*x*) respectively according to univariate GSEA. The random walk *y*_*F*1*F*2_(*x*) capturing the joint enrichment of two features can be constructed in a similar fashion. At rank *x*, *y*_*F*1*F*2_(*x*) is the number of functional genes in the union of the top *x* genes according to *F*_1_ and *F*_2_ feature scores. This procedure is illustrated in Fig. 2a. A positive deviation of *y*_*F*1*F*2_(*x*) from the diagonal line implies that the union of top-ranking genes according to *F*_1_ and *F*_2_ are enriched with the functional genes. However, multiple combinatorial relations may arise from the same enrichment outcome. Analogous to univariate GSEA, a legitimate bivariate GSEA should decipher these relations by comparing the random walks derived from single and double features.

An immediate question for bivariate GSEA is whether the two features jointly provide more enrichment information than each single feature alone. Similar procedures are found in many statistical problems such as nested model selection [11] and stepwise regression [12]. Direct comparison between the random walks of the joint features (*y*_*F*1*F*2_(*x*)) and each single feature (*y*_*F*1_(*x*) or *y*_*F*2_(*x*)) is inadequate, since *y*_*F*1*F*2_(*x*) is constructed by taking the union of two sorted gene lists, whereas *y*_*F*1_(*x*) or *y*_*F*2_(*x*) is obtained from one sorted gene list. *y*_*F*1*F*2_(*x*) thus always lies above or on *y*_*F*1_(*x*) and *y*_*F*2_(*x*) regardless of whether the joint features are more informative than each single feature or not. A fair test for the additional enrichment information of joint features *F*_1_*F*_2_ relative to a single feature *F*_1_ is to compare *y*_*F*1*F*2_(*x*) to a null model curve *y*_*F*2 ∣ *F*1_(*x*) that randomizes the enrichment outcomes of *F*_2_ conditioned on the empirical enrichment outcome of *F*_1_. More precisely, at each rank *x*, *y*_*F*2 ∣ *F*1_(*x*) counts the expected number of functional genes in the union of the top *x* genes from the sorted list according to the empirical *F*_1_ scores and the sorted list obtained by random permutations of *F*_2_ scores. The conceptual procedures of constructing a conditional random walk *y*_*F*2 ∣ *F*1_(*x*) are illustrated in Fig. 2b.

Rather than undertaking time-consuming random permutations, a conditional random walk can be evaluated analytically. At rank *n* there are *n* top-ranking genes and *k* functional genes from the *F*_1_ list. Suppose by incorporating *n* genes from a randomly sorted *F*_2_ list *n*_*extra*_ genes and *k*_*extra*_ functional genes are added. The probability that randomly selected *n* genes adds *n*_*extra*_ genes to the sorted *F*_1_ list of *n* genes is given by a hyper-geometric distribution

1 $$ {P}_{n_{extra}\mid n}=P\left({n}_{extra}\  genes\ contributed\  by\  top\ n\  genes\ in\ F2|\ F1\right)=\frac{\left(\genfrac{}{}{0pt}{}{N-n}{n_{extra}}\right)\left(\genfrac{}{}{0pt}{}{n}{n-{n}_{extra}}\right)}{\left(\genfrac{}{}{0pt}{}{N}{n}\right)} $$

The denominator denotes the number of possible combinations for choosing *n* genes according to the randomized *F*_2_ list. The two terms in the numerator denote the numbers of possible combinations for choosing *n*_*extra*_ genes outside the sorted *F*_1_ list and *n* − *n*_*extra*_ genes within the sorted *F*_1_ list.

Furthermore, conditioned on those *n*_*extra*_ genes, the probability that *k*_*extra*_ of them are functional genes is given by another hypergeometric distribution

2 $$ {P}_{k_{extra}\mid {n}_{extra}}=P\left({k}_{extra}\  cancer\ genes\  by\ F2\ |{n}_{extra}\  genes\  by\ F2\ \right)=\frac{\left(\genfrac{}{}{0pt}{}{K-k}{k_{extra}}\right)\left(\genfrac{}{}{0pt}{}{N-n-K+k}{n_{extra}-{k}_{extra}}\right)}{\left(\genfrac{}{}{0pt}{}{N-n}{n_{extra}}\right)} $$

The denominator denotes the number of possible combinations for choosing *n*_*extra*_ genes outside the sorted *F*_1_ list. The two terms in the numerator denote the numbers of possible combinations for choosing *k*_*extra*_ functional genes and *n*_*extra*_ − *k*_*extra*_ non-functional genes outside the sorted *F*_1_ list.

The expected number of extra cancer genes included in the union of the two top- *n* lists then becomes

3 $$ {y}_{F2\mid F1}(n)-{y}_{F1}(n)=\sum \limits_{n_{extra}=0}^{\min \left(n,N-n\right)}\ \sum \limits_{k_{extra}=0}^{\min \left({n}_{extra},K-k\right)}{\mathrm{P}}_{n_{extra}\mid n}\cdot {\mathrm{P}}_{k_{extra}\mid {n}_{extra}}\cdot {k}_{extra} $$

Figure 2c elucidates the relations of genes selected from the sorted *F*_1_ and *F*_2_ lists at a given rank *n*.

We compare the maximum positive deviation between the random walk of the joint features *y*_*F*1*F*2_(*x*) and the expected random walk conditioned on *F*_1_*y*_*F*2 ∣ *F*1_(*x*). A large deviation implies that *F*_2_ provides additional information about gene set enrichment after *F*_1_ is taken into account, and a small or negative deviation implies that either *F*_2_ is uninformative about gene set enrichment or its enrichment information is contained in *F*_1_. We quantify the statistical significance of the positive deviation by a one-sided Mann-Whiney U test, and use the notation *y*_*F*1*F*2_(*x*) > *y*_*F*2 ∣ *F*1_(*x*) to denote that *y*_*F*1*F*2_(*x*) significantly and positively deviates from *y*_*F*2 ∣ *F*1_(*x*), and *y*_*F*1*F*2_(*x*) ≤ *y*_*F*2 ∣ *F*1_(*x*) otherwise . Reciprocally, we compare *y*_*F*1*F*2_(*x*) and *y*_*F*1 ∣ *F*2_(*x*) to verify whether *F*_1_ provides additional enrichment information conditioned on *F*_2_.

Combining the results of univariate and bivariate GSEA, we derive the following rules for possible relations of the two features:

- *y*_*F*1_(*x*) ≤ *y*_*ϕ*_(*x*) – *F*_1_ is uninformative about gene set enrichment.
- *y*_*F*2_(*x*) ≤ *y*_*ϕ*_(*x*) – *F*_2_ is uninformative about gene set enrichment.
- *y*_*F*1_(*x*) > *y*_*ϕ*_(*x*), *y*_*F*1*F*2_(*x*) > *y*_*F*1 ∣ *F*2_(*x*), *y*_*F*1*F*2_(*x*) ≤ *y*_*F*2 ∣ *F*1_(*x*) – *F*_1_ is superior to *F*_2_ in gene set enrichment (illustrated in Additional file 1: Figure S1A).
- *y*_*F*2_(*x*) > *y*_*ϕ*_(*x*), *y*_*F*1*F*2_(*x*) > *y*_*F*2 ∣ *F*1_(*x*), *y*_*F*1*F*2_(*x*) ≤ *y*_*F*1 ∣ *F*2_(*x*) – *F*_2_ is superior to *F*_1_ in gene set enrichment.
- *y*_*F*1_(*x*) > *y*_*ϕ*_(*x*), *y*_*F*2_(*x*) > *y*_*ϕ*_(*x*), *y*_*F*1*F*2_(*x*) > *y*_*F*1 ∣ *F*2_(*x*), *y*_*F*1*F*2_(*x*) > *y*_*F*2 ∣ *F*1_(*x*) – *F*_1_ and *F*_2_ both provide indispensable enrichment information (illustrated in Additional file 1: Figure S1B).
- *y*_*F*1_(*x*) > *y*_*ϕ*_(*x*), *y*_*F*2_(*x*) > *y*_*ϕ*_(*x*), *y*_*F*1*F*2_(*x*) ≤ *y*_*F*1 ∣ *F*2_(*x*), *y*_*F*1*F*2_(*x*) ≤ *y*_*F*2 ∣ *F*1_(*x*) – *F*_1_ and *F*_2_ are largely overlapped in gene set enrichment (illustrated in Additional file 1: Figure S1C).

### Multivariate GSEA

The aforementioned procedures can be extended to *m* > 2 features. There are *m* sorted gene lists according to scores of features *F*_1_, …, *F*_*m*_ respectively. The random walk of the joint features *y*_*F*1⋯*Fm*_(*x*) is constructed by counting the functional genes in the union of *m* top- *x* gene lists. The conditional random walk ${y}_{Fi\mid F\overline{i}}(x)$ is obtained by fixing *m* − 1 top-ranking gene lists from features ${F}_{\overline{i}}\equiv \left\{{F}_1,\cdots, {F}_{i-1},{F}_{i+1},\cdots, {F}_m\right\}$ and randomly permuting the gene list from feature *F*_*i*_. ${y}_{Fi\mid F\overline{i}}(x)$ can be calculated with the same formulas of equations 1, 2 and 3 by substituting the conditioned features ${F}_{\overline{i}}$ for *F*_1_. In principle, one can construct a conditional random walk by permuting the scores of an arbitrary subset of features and fixing all the remaining ones. However, the union of multiple permuted gene lists gives rise to very complicated inclusion-exclusion relations and cannot be reduced to simple forms like equations 1, 2 and 3. Therefore, we only allow the conditional random walks with one feature subjected to random permutations (e.g., *y*_*F*1 ∣ *F*2*F*3_(*x*)), and discard all the remaining conditional random walks (e.g., *y*_*F*2*F*3 ∣ *F*1_(*x*)).

More combinatorial relations of gene set enrichment will also arise when multiple features are considered. Yet these combinatorial relations can be reduced to two simple rules according to multivariate joint and conditional random walks. We define a feature *dominant* among a collection of features if its gene set enrichment information is not subsumed to any other subset of features. Likewise, a subset of features are *redundant* if they carry significant gene set enrichment information but their information is largely overlapped. We adopt the following rules to determine whether a feature is dominant or whether two features are redundant:

- *F*_1_ is dominant if *y*_*F*1_(*x*) > *y*_*ϕ*_(*x*) and *y*_*F*1*FI*_(*x*) > *y*_*F*1 ∣ *FI*_(*x*) for all subsets of features *F*_*I*_ that do not contain *F*_1_.
- *F*_1_ and *F*_2_ are redundant if *y*_*F*1_(*x*) > *y*_*ϕ*_(*x*), *y*_*F*2_(*x*) > *y*_*ϕ*_(*x*), *y*_*F*1*F*2*FI*_(*x*) ≤ *y*_*F*1 ∣ *F*2*FI*_(*x*), *y*_*F*1*F*2*FI*_(*x*) ≤ *y*_*F*2 ∣ *F*1*FI*_(*x*) for all subsets of features *F*_*I*_ that do not contain *F*_1_ and *F*_2_.

Redundant relations are transitive: if *F*_1_ and *F*_2_ are redundant and *F*_2_ and *F*_3_ are redundant, then *F*_1_ and *F*_3_ are redundant. The aforementioned combinatorial rules of bivariate GSEA can also be simplified in terms of dominance and redundancy of features. Condition 1: *F*_1_ is not dominant. Condition 2: *F*_2_ is not dominant. Condition 3: *F*_1_ is dominant. Condition 4: *F*_2_ is dominant. Condition 5: *F*_1_ and *F*_2_ are dominant. Condition 6: *F*_1_ and *F*_2_ are redundant.

## Results

We justified the utility of MGSEA by four studies. First, we simulated feature scores and gene set memberships according to several combinatorial relations and demonstrated that MGSEA could recover these relations. Second, we defined feature scores of multimodal cancer OMIC data (CNV, DNA methylation, mRNA expression) in terms of their capabilities to delineate tumor subtypes and applied MGSEA to the breast cancer and glioblastoma multiforme (GBM) data from The Cancer Genome Atlas (TCGA). Analysis results indicated that mRNA expression was a dominant feature in many functional categories of both cancer types, and CNV was a dominant feature in many functional categories of breast cancer. Third, we validated these combinatorial relations by applying MGSEA to external breast cancer and GBM data. Analysis results derived from external data were substantially compatible with those derived from TCGA. Fourth, we compared MGSEA with several integrative methods of gene set enrichment by both listing the common and distinct characteristics for each method and quantitatively contrasting their data analysis outcomes.

### Analysis from simulated data

We generated random scores of 1000 genes on 3 features (*x*_1_, *x*_2_, *x*_3_) and created binary indicators (*y*) for the gene set membership. Feature scores were sampled from a uniform distribution over [0, 1]. Four models were employed to specify the relation between (*x*_1_, *x*_2_, *x*_3_) and *y*: (1) *y* was sampled from logistic regression $P\left(y=1|{x}_1,{x}_2,{x}_3\right)=\frac{\exp \left(20{x}_1\right)}{1+\exp \left(20{x}_1\right)}$, (2) $P\left(y=1|{x}_1,{x}_2,{x}_3\right)=\frac{\exp \left(20\left({x}_1+{x}_2\right)\right)}{1+\exp \left(20\left({x}_1+{x}_2\right)\right)}$, (3) $P\left(y=1|{x}_1,{x}_2,{x}_3\right)=\frac{\exp \left(20\left({x}_1+{x}_2+{x}_3\right)\right)}{1+\exp \left(20\left({x}_1+{x}_2+{x}_3\right)\right)}$, (4) *z* was uniformly sampled over [0, 1], $P\left(y=1|z\right)=\frac{\exp (20z)}{1+\exp (20z)}$, and *x*_1_ = *t*_[0, 1]_(*z* + *e*_1_), *x*_2_ = *t*_[0, 1]_(*z* + *e*_2_), where *t*_[0, 1]_(.) is a truncation function that sets values >1 to 1 and values <0 to 0, and *e*_1_, *e*_2_~*N*(0,0.1). In brief, models 1–3 specify that *x*_1_, *x*_1_*x*_2_, and *x*_1_*x*_2_*x*_3_ are the dominant features respectively, and model 4 specifies that *x*_1_ and *x*_2_ are redundant features.

**Fig. 3:**
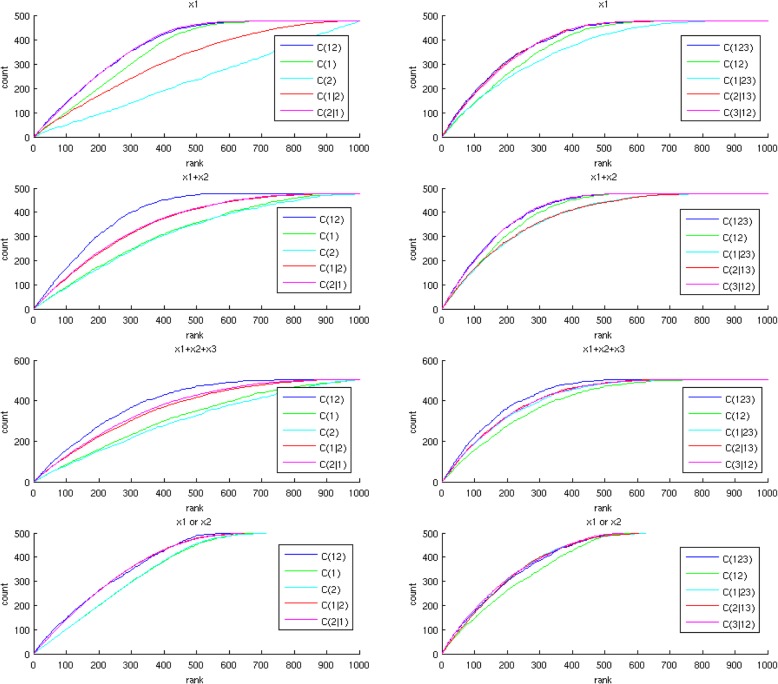
GSEA random walks of simulated data generated from four models. Each row shows the results from one model. The left and right columns display the random walks pertaining to two features (*x*_1_ and *x*_2_) and three features (*x*_1_, *x*_2_ and *x*_3_) respectively

Figure 3 displays the random walks of two features (the left column) and three features (the right column) for the four models (four rows). For model 1 (the first row), the univariate random walk of *x*_1_ (*C*(1), the left column) is superior to the null model (the undisplayed diagonal line), the univariate random walk of *x*_2_ (*C*(2)) is not superior to the null model, the joint random walk of *x*_1_*x*_2_ (*C*(12)) is superior to the conditional random walk given *x*_2_ (*C*(1| 2)), but is not superior to the conditional random walk given *x*_1_ (*C*(2| 1)), indicating *x*_1_ is superior to *x*_2_ in gene set enrichment. The joint random walk of *x*_1_*x*_2_*x*_3_ (*C*(123), the right column) is superior to the conditional random walk given *x*_2_*x*_3_ (*C*(1| 23)), but is not superior to the conditional random walks given *x*_1_*x*_3_ (*C*(2| 1 3)) and *x*_1_*x*_2_ (*C*(3| 12)), indicating again that *x*_1_ is the only dominant feature. For model 2 (the second row), both *C*(1) and *C*(2) are superior to the null model, and *C*(12) is superior to both *C*(1| 2) and *C*(2| 1), indicating that both *x*_1_ and *x*_2_ provide indispensable enrichment information. *C*(123) is superior to *C*(1| 23) and *C*(2| 13), but is not superior to *C*(3| 12), suggesting that *x*_3_ is uninformative of gene set enrichment given *x*_1_ and *x*_2_. For model 3 (the third row), the random walks pertaining to two features *x*_1_ and *x*_2_ (the left panel) are similar to those of model 2. *C*(123) is superior to *C*(1| 23), *C*(2| 13), and *C*(3| 12), indicating that *x*_1_, *x*_2_ and *x*_3_ all provide indispensable information in gene set enrichment. For model 4 (the fourth row), both *C*(1) and *C*(2) are superior to the null model, but *C*(12) is not superior to either *C*(1| 2) or *C*(2| 1), indicating that *x*_1_ and *x*_2_ provide redundant information about gene set enrichment. The random walks pertaining to three features suggest that no feature is dominant.

### Analysis from TCGA trimodal data of breast cancer and glioblastoma patients

We further employed MGSEA to analyze the integrated OMIC data from the TCGA database. The goal of this analysis was to (1) identify the informative markers in each platform that distinguish tumor subtypes, (2) find the functional gene sets enriched with these informative markers, (3) for each selected gene set infer the combinatorial relations of enrichment information among the platforms, (4) deduce the patterns of those combinatorial relations from all selected gene sets. Two cancer types – breast cancer [2] and glioblastoma multiforme [3] were selected. For each cancer type, we downloaded the data of CNV (CNV-SNP microarrays), DNA methylations (450 K BeadChip), and mRNA expressions (microarrays and RNASeq). 340 breast cancer samples and 63 GBM samples possess all three types of data with sporadic missing values.

The level-2 data downloaded from the TCGA database were converted into a standard format with the following procedures [13]. First, probe-level data (CNV, mRNA microarray) and gene-level data (RNASeq) were rank-transformed into CDF values for each probe/gene separately. The normalized CDF values fell in the range [0, 1] and reflected the relative orders of feature values. For CNV data, the normalized CDF values were adjusted to reduce over-estimation of amplification and deletion events. DNA methylation data did not need normalization as their outputs (*β* values) were already in [0, 1]. Second, probe-level data were converted into gene-level data by averaging over the probe values for each gene. Third, we filtered out the genes whose feature values were dominated by either missing entries or zeros (more than half of the samples possess invalid values). For breast cancer, the processed data covered 21,501 genes for CNV, 13933 genes for DNA methylations and 20,764 genes for mRNA expressions; while for GBM, the corresponding numbers of genes were 21,491, 14,307, and 19,024 respectively. 10,400 and 10,562 genes possessed all three types of data for breast cancer and GBM, respectively.

As a proof-of-concept demonstration, we chose a well-known task of delineating cancer subtypes with CNV, DNA methylation and mRNA expression data. There are four breast cancer subtypes – basal-like, luminal A, luminal B, and HER2-enriched [14], and four GBM subtypes – classical, neural, proneural, and mesenchymal [15]. For each feature, we defined a gene score as the mutual information between subtype labels and feature values (CNV level, DNA methylation level, or mRNA expression level) of a gene over the samples:

4 $$ I\left(X;Y\right)=\sum \limits_{i=1}^4P\left(y=i\right)\int p\left(x|y=i\right)\log \left(\frac{p\left(x|y=i\right)}{p(x)}\right) dx $$

*X* and *Y* denote feature values and subtype labels respectively. *X* is a continuous random variable, and its marginal probability density function (*p*(*x*)) and conditional probability density function (*p*(*x* ∣ *y*)) were inferred from kernel density estimation. *Y* is a discrete random variable, and its probability mass function (*P*(*y*)) was empirically estimated by counting the fraction of samples belonging to each subtype. The mutual information score captures the dependency of subtype labels and feature values for each gene.

It is curious to know whether the data of each platform provides indispensable information about cancer subtype delineation or the information from some platforms is redundant given those from other platforms. To uncover the correlation structure of information from multiple platforms, we sorted genes in terms of the mutual information scores from one platform (e.g., CNV) and compared the distributions of the mutual information scores from another platform (e.g., mRNA expression) between the top-ranking genes and all the genes. Additional file 2: Figure S2 displays the comparison results for all pairs of platforms. Overall, there is low correlation between the information from distinct platforms, as the mutual information scores of one platform are not significantly different between the top-ranking genes and all the genes in terms of the mutual information scores of another platform.

The purpose of gene set enrichment in this task is to find the functional categories of genes that are informative about the cancer subtypes. For each cancer type, we sorted genes in a decreasing order according to their mutual information scores of each platform separately and selected the union of top-ranking genes from all 3 platforms so that 5000 valid genes were included in the universe gene set. We solicited Gene Ontology (GO) categories (http://www.geneontology.org/) [16, 17] that contained at least 50 genes in the universe gene set (resulting in 1073 and 1099 gene sets for breast cancer and GBM, respectively) and 50 hallmark gene sets from MSigDB [1, 18]. Both Gene Ontology and Hallmark gene sets were downloaded from the Molecular Signatures Database (MSigDB) (http://software.broadinstitute.org/gsea/msigdb). We then performed univariate and multivariate GSEA on those functional categories. This requires evaluations of equations 1, 2 and 3 at 5000 ranks over 2172 gene sets. To reduce computation time, we down-sampled the ranks by ten folds, evaluated the random walk displacements at 500 equally distanced “knot” ranks, and constructed a piecewise linear function connecting the knot values as the approximated random walk. Denote features 1, 2 and 3 as CNV, DNA methylation, and mRNA expression respectively. The Mann-Whitney *p*-values of 16 comparisons of GSEA random walks were reported: *C*(1) vs *C*(*ϕ*), *C*(2) vs *C*(*ϕ*), *C*(3) vs *C*(*ϕ*), *C*(12) vs *C*(*ϕ*), *C*(23) vs *C*(*ϕ*), *C*(13) vs *C*(*ϕ*), *C*(123) vs *C*(*ϕ*), *C*(12) vs *C*(1| 2), *C*(12) vs *C*(2| 1), *C*(23) vs *C*(2| 3), *C*(23) vs *C*(3| 2), *C*(13) vs *C*(1| 3), *C*(13) vs *C*(3| 1), *C*(123) vs *C*(1| 23), *C*(123) vs *C*(2| 13), *C*(123) vs *C*(3| 12).

To judge whether each comparison gave rise to a significant positive deviation, we set the threshold of Mann-Whitney p-values to 10^−10^ and labeled a comparison significant if the p-value was ≤ the threshold. The threshold was determined by the following procedures. For any given *p*-value cutoff, we calculated the false discovery rate (FDR) for detecting significantly enriched gene sets. From the empirical data, we assessed the *p*-values of univariate GSEA for all gene sets and counted the number of significantly enriched gene sets according to the given *p*-value threshold. We then randomly permuted the mutual information scores of the genes 1000 times. In each random trial, the number of significantly enriched gene sets was counted in the same fashion. The FDR was the expected number of significantly enriched gene sets arising from randomized data divided by the number of significantly enriched gene sets derived from the empirical data:

$$ False\ Discovery\ Rate=\min \left(\frac{\mathrm{total}\#\mathrm{Signficant}\ \mathrm{gene}\ \mathrm{sets}\ \mathrm{in}\ \mathrm{the}\ \mathrm{permuted}\ \mathrm{data}}{\#\mathrm{Significant}\ \mathrm{gene}\ \mathrm{sets}\ \mathrm{in}\ \mathrm{the}\ \mathrm{empirical}\ \mathrm{data}\times 1000},1\right) $$

FDR according to this definition is a function of the *p*-value threshold. Additional file 3: Figure S3 shows the FDRs for the three feature scores in TCGA breast cancer and GBM data (the left column). The FDRs of all features generally declined with decreasing p-value thresholds. In breast cancer, at the p-value cutoff 10^− 10^, the FDRs of both mRNA and CNV were around 0.4, while DNA methylation had a considerably higher FDR (around 0.7). In GBM, at the same p-value cutoff the FDRs of mRNA, DNA methylation, and CNV were about 0.2, 0.5, and 0.8 respectively.

The poor FDRs for DNA methylation in both cancers and CNV in GBM data indicate that the top-ranking genes in terms of these feature scores are enriched with fewer functional gene sets. We selected the top 100 genes in terms of each feature score and counted the number of significantly enriched gene sets according to the Fisher exact test (p-value cutoff 0.05, Additional file 4: Table S1). Indeed, the number of significantly enriched gene sets according to mRNA expressions was substantially higher than those according to CNV and DNA methylation in GBM data, and comparable to CNV in breast cancer data.

#### Functional enrichment of breast cancer subtype biomarkers

434 functional categories contained at least one dominant feature or one pair of redundant features in the breast cancer enrichment outcomes. CNV, DNA methylation and mRNA expression were dominant in 147, 137 and 179 functional categories respectively. (CNV, DNA methylation), (DNA methylation, mRNA expression), and (CNV, mRNA expression) pairs were dominant in 3, 8 and 18 functional categories respectively. Many functional categories either were highly overlapped or had nested subsumption relations. The GO terms from breast cancer data were summarized using REVIGO [19] and were reduced into 212 groups. The parameter setting of running REVIGO is reported in Additional file 5: Table S2. The Mann-Whitney *p*-values of all 16 pairwise random walk comparisons among the 434 functional categories are reported in Additional file 6: Table S3. The combinatorial relations of the three features in the 434 functional categories are reported in Additional file 7: Table S4 and the combinatorial relations of the three features in the 212 reduced functional categories are reported in Table 1.

**Table 1:** Combinatorial relations of enrichment information in 126 reduced functional classes of breast cancer data

Combinatorial Pattern							Gene set
CNV	MET	MRNA	CNV and MET	MET and MRNA	CNV and MRNA	CNV and MET and MRNA	
0	0	1	0	0	0	0	Carbohydrate biosynthetic process, Cellular response to DNA damage stimulus, Chromatin remodeling, Chromosome, Chromosome organization, DNA recombination, Epidermis development, Extracellular matrix, Heparin binding, Microtubule based movement, Morphogenesis of a branching structure, Nuclear chromosome segregation, Organic acid catabolic process, Pallium development, Positive regulation of growth, Regulation of neuron apoptotic process, Regulation of protein complex disassembly, Response to purine containing compound, Response to radiation, Second messenger mediated signaling, Sex differentiation, Signal release, Supramolecular fiber, Tubulin binding, Aminoglycan metabolic process, Anatomical structure homeostasis, Apical plasma membrane,Cell cycle, Cell division, Cell proliferation, Cellular response to acid chemical, Chromosome segregation, Digestive system development, DNA metabolic process, Gland development, Growth, Lyase activity, Mammary gland development, Microtubule based process, Midbody, Negative regulation of locomotion, Nuclear membrane, Organelle localization, Ossification, Protein homodimerization activity, Regulation of cell division, Regulation of ligase activity, Regulation of neurotransmitter levels, Regulation of ossification, Regulation of transmembrane receptor protein serine threonine kinase signaling pathway, Response to drug, Response to ketone, Response to toxic substance, Response to transition metal nanoparticle, Stem cell differentiation, Tube development, Apical surface, DNA repair, E2F targets, Estrogen response early, Estrogen response late, Fatty acid metabolism, G2M checkpoint, Glycolysis, Hedgehog signaling, Hypoxia, Mitotic spindle, MTORC1 signaling, MYC targets v1,MYC targets v2,Peroxisome, Spermatogenesis
0	1	0	0	0	0	0	Apoptotic signaling pathway, Cell substrate adhesion, Central nervous system neuron differentiation, Core promoter binding, ER to Golgi vesicle mediated transport, Interaction with host, Macromolecular complex disassembly, Negative regulation of phosphorylation, Peptidase inhibitor activity, Peptidyl Serine modification, Protein catabolic process, RAS protein signal transduction, Regulation of binding, Regulation of protein import, Response to carbohydrate, Response to endoplasmic reticulum stress, Small molecule biosynthetic process, Transcription corepressor activity, Transferase complex, Ubiquitin like protein ligase binding, WNT signaling pathway, Actin filament organization, Aging, Binding bridging, Cell cortex, Cell junction assembly, Cell junction organization, Cellular carbohydrate metabolic process, Cellular component disassembly, Cellular response to abiotic stimulus, Coenzyme binding, Cofactor binding, Cytoplasmic region, Energy derivation by oxidation of organic compounds, Establishment or maintenance of cell polarity, Heart morphogenesis, Hormone receptor binding, In utero embryonic development, Ligase activity, Lytic vacuole membrane, Macromolecule methylation, Mitochondrial matrix, Myelin sheath, Placenta development, Protein folding, Protein stabilization, Regulation of autophagy, Regulation of gene expression epigenetic, Regulation of protein stability, Regulation of response to extracellular stimulus, Regulatory region nucleic acid binding, RNA splicing, Transcription factor activity protein binding, Transcription factor binding, Transcription factor complex, Ubiquitin like protein transferase activity, Vacuole organization, Adipogenesis, Angiogenesis, Cholesterol homeostasis, Coagulation, Complement, Oxidative phosphorylation, TGF beta signaling, Unfolded protein response
0	1	1	0	1	0	0	Positive regulation of apoptotic signaling pathway, Magnesium ion binding, Negative regulation of protein serine threonine kinase activity, Positive regulation of cellular protein localization, Signal transduction by p53 class mediator, Telencephalon development, Notch signaling
1	0	0	0	0	0	0	Adaptive immune response, Anion transport, Cell-cell adhesion via plasma membrane adhesion molecules, Clathrin coated vesicle, Cognition, Excitatory synapse, Formation of primary germ layer, Growth factor receptor binding, GTPase activity, Hormone mediated signaling pathway, Muscle cell differentiation, Organic acid transmembrane transporter activity, Organic cyclic compound catabolic process, RAS guanyl nucleotide exchange factor activity, Regulation of body fluid levels, Regulation of cytokine production, Regulation of ion homeostasis, Regulation of stat cascade, Ribosome biogenesis, Transcriptional repressor activity RNA polymerase II transcription regulatory region sequence specific binding, Wound healing, Anterior posterior pattern specification, Cardiac chamber development, Cation channel complex, Cell activation, Cell adhesion molecule binding, Cell-cell signaling, Cell fate commitment, Cell junction, Cytosolic transport, G protein coupled receptor signaling pathway coupled to cyclic nucleotide second messenger, Intermediate filament cytoskeleton, Multi organism reproductive process, Muscle structure development, Muscle tissue development, Negative regulation of response to external stimulus, Organic acid transport, Receptor complex, Regulation of response to biotic stimulus, Regulation of transporter activity, Respiratory system development, Ribosome, rRNA metabolic process, Single organism behavior, Site of polarized growth, Skeletal system development, Synaptic signaling, Transmembrane receptor protein serine threonine kinase signaling pathway, Transporter complex, Androgen response, Epithelial mesenchymal transition, Il6 JAK STAT3 signaling, Pancreas beta cells, Reactive oxygen species pathway
1	0	1	0	0	1	0	Kidney epithelium development, Meiotic cell cycle process, Response to alcohol, Voltage gated ion channel activity, Lipid modification, Nuclear periphery, Positive regulation of cell division, Potassium ion transport, Regulation of organ morphogenesis, Urogenital system development, Bile acid metabolism
1	1	0	1	0	0	0	Core promoter proximal region DNA binding, Response to nutrient, RNA polymerase II transcription factor activity sequence specific DNA binding

CNV, DNA methylation, and mRNA expression appeared in single dominant or dominant combinatorial relations in 68, 75 and 90 reduced functional categories respectively, indicating informative marker genes in terms of mRNA expression were moderately more enriched with known functional categories than CNV and DNA methylation. About 90% of the reduced functional categories possessed one dominant feature: 54, 65, 72 for CNV, DNA methylation, and mRNA expression respectively. In contrast, only a small number of reduced functional categories possessed multiple dominant features: 3, 7, 11 for CNV-DNA methylation, DNA methylation-mRNA expression, and CNV-mRNA expression pairs respectively.

Many reduced functional categories appeared in Table 1 were involved in well-known cancer-related processes. Furthermore, functional categories belonging to different combinatorial patterns tended to concentrate on distinct underlying processes. For instance, many reduced functional categories involved in cell proliferation (e.g., cell cycle control, epithelial cell development, *MYC* targets, *E2F* targets, estrogen response, and DNA repair) possessed mRNA expression as the only dominant feature. In contrast, several reduced functional categories involved in cell invasion and metastasis (e.g., cell adhesion, epithelial-mesenchymal transition (EMT), and immune response) possessed CNV as the only dominant feature. Positive regulation of cell division possessed mRNA expression and CNV as the dominant features; Notch signaling and TP53 signaling possessed mRNA expression and DNA methylation as the dominant features.

**Fig. 4:**
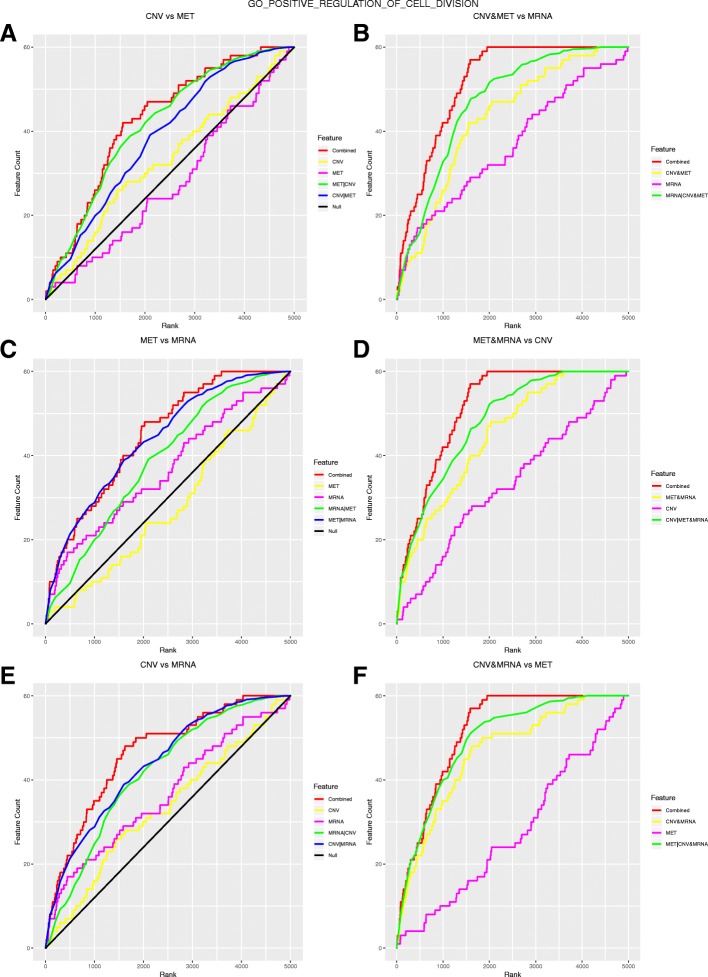
MGSEA random walks of positive regulation of cell division on breast cancer data. Panels A, C, E display comparisons of two features with the corresponding conditional random walks. Panels B, D, F display comparisons of three features with the corresponding random walks. Each row shows the comparison results of distinct combinations of the features

We illustrate the interpretation of the MGSEA outcomes with a functional category of positive regulation of cell division. It possessed the dominant features of CNV and mRNA expression. Figure 4 shows the MGSEA random walks of positive regulation of cell division. When comparing the joint random walks of two features with the corresponding conditional random walks (the left column), we found that *C* (CNV,MRNA) (Fig. 4e, red) was superior to both *C* (CNV|MRNA) (blue) and *C* (MRNA|CNV) (green), while *C* (CNV,MET) (Fig. 4a, red) was superior to *C* (CNV|MET) (blue) but not superior to *C* (MET|CNV) (green), and *C* (MET,MRNA) (Fig. 4c, red) is superior to *C* (MRNA|MET) (green) but not superior to *C* (MET|MRNA) (blue). The results indicated that the enrichment information of DNA methylation was subsumed to both CNV and mRNA expression, while CNV and mRNA expression were both indispensable. Comparison of the joint random walks of three features with the corresponding conditional random walks (the right column) also corroborated this conclusion. *C* (CNV,MET,MRNA) (Fig. 4f, red) was not superior to *C* (MET|CNV,MRNA) (green), suggesting that randomizing DNA methylation did not lose extra information. In contrast, *C* (CNV,MET,MRNA) was superior to both *C* (MRNA|CNV,MET) (Fig. 4b, green) and *C* (CNV|MET,MRNA) (Fig. 4d, green), suggesting that CNV and mRNA expression provided indispensable enrichment information.

**Fig. 5:**
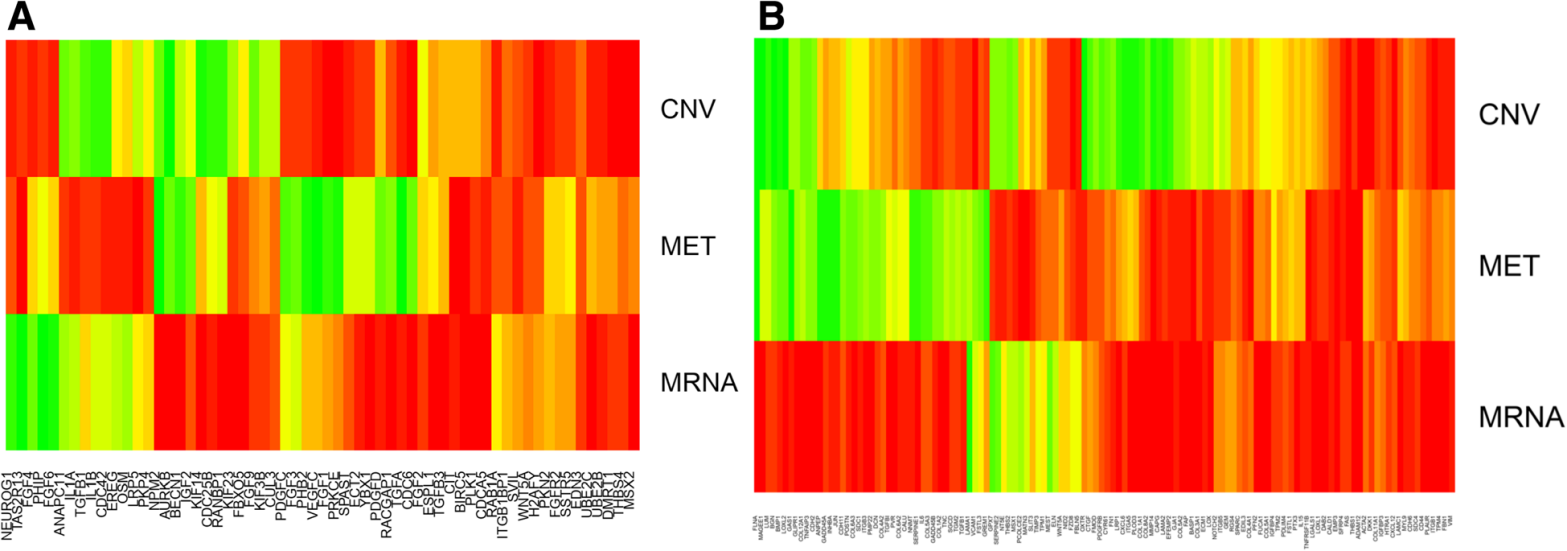
Mutual information scores of three features on (A) Positive regulation of cell division on breast cancer data, and (B) EMT on GBM data. Warm colors indicate high values. Top: CNV, middle: DNA methylation, bottom: mRNA expression

The combinatorial relations of features can also be revealed in their mutual information scores. Figure 5a displays the mutual information scores of three features on positive regulation of cell division. High-scoring genes in terms of CNV and mRNA expression were not highly overlapped. In contrast, high-scoring genes in terms of DNA methylation were mostly contained in high-scoring genes in terms CNV and mRNA expression. Therefore, both CNV and mRNA expression were dominant and DNA methylation is subsumed to them.

#### Functional enrichment of glioblastoma subtype biomarkers

676 functional categories contained at least one dominant feature or one pair of redundant features in the GBM enrichment outcomes. We again performed REVIGO analysis on the membership vectors of these functional categories and reduced them to 272 groups. The Mann-Whitney *p*-values of 16 pairwise random walk comparisons among the 676 functional categories are reported in Additional file 8: Table S5. The combinatorial relations of the three features in the 676 functional categories are reported in Additional file 9: Table S6 and the combinatorial relations of the three features in the 272 reduced functional categories are reported in Table 2.

**Table 2:** Combinatorial relations of enrichment information in 128 reduced functional classes of GBM data

Combinatorial Pattern							Gene set
CNV	MET	MRNA	CNV and MET	MET and MRNA	CNV and MRNA	CNV and MET and MRNA	
0	0	1	0	0	0	0	Actin based cell projection, Actin cytoskeleton, Antigen processing and presentation, Apoptotic signaling pathway, Bone development, Carbohydrate derivative catabolic process, Cell cycle arrest, Cell growth, Cell junction assembly, Cell substrate adhesion, Cell substrate junction, Cellular response to external stimulus, Cellular response to nitrogen compound, Coated vesicle, Covalent chromatin modification, Cytoplasmic side of membrane, Endocytosis, Endoplasmic reticulum lumen, Interaction with host, Membrane organization, Morphogenesis of a branching structure, Negative regulation of transferase activity, Organelle localization, Organic acid biosynthetic process, Poly a RNA binding, Positive regulation of cell proliferation, Positive regulation of cytoplasmic transport, Protein autophosphorylation, Protein dephosphorylation, Protein heterodimerization activity, Regulation of cell activation, Regulation of ossification, Regulation of peptide secretion, Regulation of protein stability, Regulation of synapse organization, Regulation of synaptic plasticity, Response to carbohydrate, Response to temperature stimulus, Second messenger mediated signaling, Somatodendritic compartment, Telencephalon development, Transferase activity transferring glycosyl groups, Transmembrane receptor protein tyrosine kinase signaling pathway, Tubulin binding, Ubiquitin like protein ligase binding, Vacuolar lumen, Vesicle membrane, Vesicle organization, Wound healing, Actin filament based process, Aging, Autophagy, Binding bridging, Biological adhesion, Calmodulin binding, Carbohydrate binding, Cell activation, Cell adhesion molecule binding, Cell body, Cell cortex, Cell death, Cell junction, Cell junction organization, Cell leading edge, Cell projection, Cell surface, Chromatin modification, Circulatory system development, Connective tissue development, Cysteine type peptidase activity, Cytokine production, Cytoplasmic region, Cytoskeletal protein binding, Endomembrane system organization, Enzyme binding, Excitatory synapse, Extracellular matrix component, Extracellular structure organization, Glycosaminoglycan binding, Growth, Growth factor binding, Homeostasis of number of cells, Identical protein binding, Immune system process, Kinase regulator activity, Locomotion, Macromolecular complex binding, Movement of cell or subcellular component, Multicellular organism metabolic process, Nuclear body, Organelle subcompartment, Ossification, Perinuclear region of cytoplasm, Plasma membrane organization, Positive regulation of proteolysis, Posttranscriptional regulation of gene expression, Protein complex binding, Protein dimerization activity, Protein domain specific binding, Protein folding, Receptor binding, Receptor complex, Receptor signaling protein activity, Regeneration, Regulation of autophagy, Regulation of gene expression epigenetic, Regulation of reactive oxygen species metabolic process, Regulation of stem cell differentiation, Regulation of synapse structure or activity, Respiratory system development, Response to biotic stimulus, Response to transition metal nanoparticle, Sarcolemma, Sh3 domain binding, Side of membrane, Site of polarized growth, Sphingolipid metabolic process, Sulfur compound biosynthetic process, Sulfur compound metabolic process, Symporter activity, Trans Golgi network, Transcription coactivator activity, Ubiquitin ligase complex, Vacuole, Allograft rejection, Angiogenesis, Apical junction, Apical surface, Apoptosis, Cholesterol homeostasis, Coagulation, Complement, Epithelial mesenchymal transition, G2M checkpoint, Hedgehog signaling, Hypoxia, Il2 stat5 signaling, Il6 JAK STAT3 signaling, Inflammatory response, Interferon gamma response, KRAS signaling up, Mitotic spindle, Myogenesis, P53 pathway, TNFa signaling via NFkB, Unfolded protein response, UV response down
0	1	0	0	0	0	0	Anion transmembrane transport, Calcium ion transmembrane transporter activity, Cellular response to inorganic substance, Cilium, Epidermis development, Hindbrain development, Negative regulation of cell growth, Negative regulation of secretion, Nephron development, Post synapse, RAS guanyl nucleotide exchange factor activity, Regulation of lipid biosynthetic process, Regulation of muscle cell differentiation, Regulation of reproductive process, Response to ketone, Sensory perception of mechanical stimulus, Signaling receptor activity, Skeletal system morphogenesis, Ameboidal type cell migration, Amide biosynthetic process, Ammonium ion metabolic process, Anchored component of membrane, Cation channel complex, Cell maturation, G protein coupled receptor signaling pathway, Growth factor activity, GTPase binding, Intrinsic component of plasma membrane, Membrane protein complex, Membrane region, Mitochondrial transport, Multi multicellular organism process, Multicellular organismal signaling, Pattern specification process, Positive regulation of cell division, Regionalization, Regulation of camp metabolic process, Regulation of membrane potential, Regulation of transmembrane receptor protein serine threonine kinase signaling pathway, Response to estrogen, Response to light stimulus, Response to starvation, Stem cell differentiation, Structural molecule activity, Transporter complex, Urogenital system development, UV response up
0	1	1	0	1	0	0	Cell part morphogenesis, Cytokine receptor binding, Extrinsic component of plasma membrane, Inositol lipid mediated signaling, Negative regulation of nervous system development, Regulation of epithelial cell proliferation, Regulation of wound healing, Vasculature development, Extracellular matrix, Extrinsic component of membrane, Membrane microdomain, Peptidyl tyrosine modification, Positive regulation of peptidase activity, Protein tyrosine kinase activity, Regulation of chemotaxis, Regulation of extrinsic apoptotic signaling pathway, Regulation of receptor activity, Synapse organization, Interferon alpha response, Notch signaling, WNT beta catenin signaling
1	0	0	0	0	0	0	Cell cycle phase transition, Chromosomal region, Circadian rhythm, Hexose metabolic process, MRNA metabolic process, Negative regulation of cellular catabolic process, Negative regulation of hydrolase activity, Peptidase inhibitor activity, Positive regulation of DNA metabolic process, Positive regulation of homeostatic process, Regulation of cation transmembrane transport, Regulation of protein modification by small protein conjugation or removal, Regulatory region nucleic acid binding, RNA splicing via transesterification reactions, Sex differentiation, Adenylate cyclase modulating g protein coupled receptor signaling pathway, B cell activation, Coenzyme metabolic process, Cofactor metabolic process, Cytosolic part, Endocrine system development, Hemostasis, Hepaticobiliary system development, Hormone metabolic process, Hormone receptor binding, Iron ion binding, Lipid localization, Metallopeptidase activity, Methylation, ncRNA metabolic process, Nucleic acid binding transcription factor activity, Organic hydroxy compound biosynthetic process, Oxidoreductase activity acting on paired donors with incorporation or reduction of molecular oxygen, Positive regulation of WNT signaling pathway, RAS protein signal transduction, Response to calcium ion, Response to drug, Rhythmic process, Tetrapyrrole binding, Transcription factor complex, Transferase activity transferring acyl groups, Transferase complex transferring phosphorus containing groups, Bile acid metabolism, Protein secretion
1	0	1	0	0	1	0	Adaptive immune response based on somatic recombination of immune receptors built from immunoglobulin superfamily domains, Positive regulation of binding, Regulation of endocytosis, Negative regulation of cell-cell adhesion, Negative regulation of cellular response to growth factor stimulus, Phosphatase binding, Regulation of leukocyte proliferation
1	1	0	1	0	0	0	Cell fate commitment, G protein coupled receptor signaling pathway coupled to cyclic nucleotide second messenger, Mesenchymal cell differentiation, Negative regulation of canonical WNT signaling pathway
1	1	1	1	1	1	1	Pancreas beta cells, TGF beta signaling

Unlike breast cancer data, the majority of the functional categories (and reduced functional categories) were dominated by mRNA expression: CNV, DNA methylation and mRNA expression were dominant in 92, 150 and 493 functional categories and 57, 74 and 177 reduced functional categories. The top 4 most abundant combinatorial relations were mRNA expression dominant (147 reduced functional categories), DNA methylation dominant (47 reduced functional categories), CNV dominant (44 reduced functional categories), and DNA methylation and mRNA expression dominant (23 reduced functional categories). All the other combinatorial relations were rare.

The reduced functional categories possessing mRNA expression as a dominant feature were quite different between breast cancer and GBM data. There were 72 and 147 such reduced functional categories in breast cancer and GBM data respectively, and only 8 of them appeared in both datasets. In GBM data, these reduced functional categories were involved in distinct cancer-related processes from breast cancer data, such as angiogenesis, cell-cell adhesion, immune response, inflammatory response, and EMT. The reduced functional categories that appeared in both datasets included mitotic spindle, apical surface, Hedgehog signaling, hypoxia, and G2M checkpoint.

**Fig. 6:**
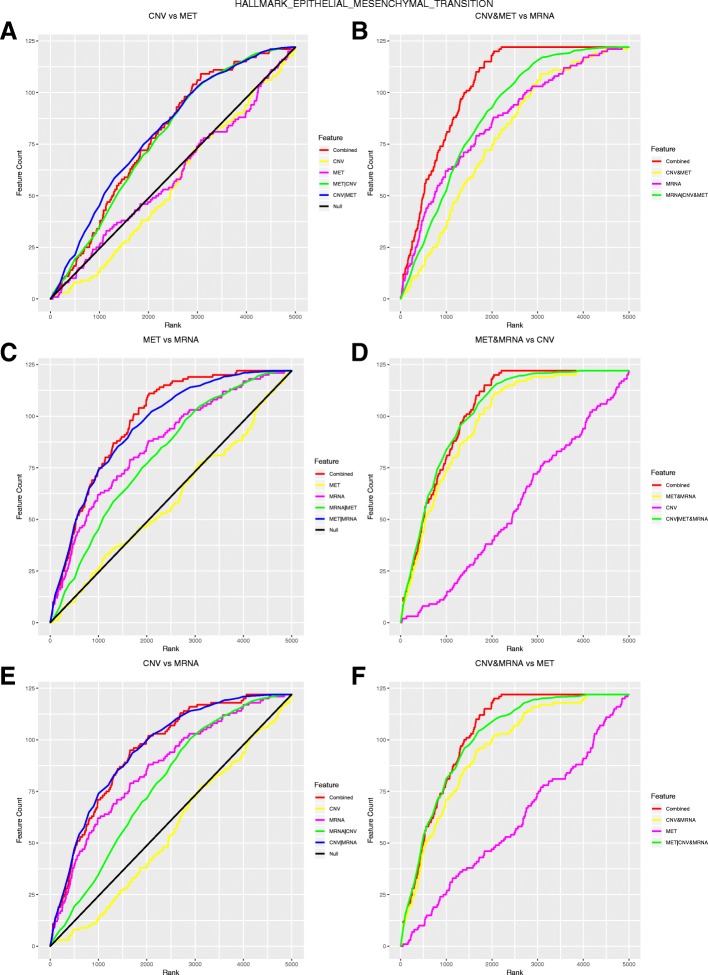
MGSEA random walks of Epithelial Mesenchymal Transition on GBM data. Panels A, C, E display comparisons of two features with the corresponding conditional random walks. Panels B, D, F display comparisons of three features with the corresponding random walks. Each row shows the comparison results of distinct combinations of the features

We also illustrate the interpretation of the MGSEA outcomes with a functional category of EMT. Figure 6 shows the MGSEA random walks pertaining to two and three features of EMT. The random walks of the joint features including mRNA expression (e.g., *C* (MET,MRNA), Fig. 6c, red) were superior to the conditional random walks randomizing mRNA expression (e.g., *C* (MRNA|MET), Fig. 6c, green), indicating the dominance of mRNA expression. In contrast, CNV and DNA methylation were both subsumed to mRNA expression. The dominance of mRNA expression was also manifested in the mutual information scores in Fig. 5b. High-scoring genes were populated in mRNA expression, and the high-scoring genes in CNV and DNA methylation scores were overlapped with the high-scoring genes in mRNA expression scores.

### Validation with external datasets

To verify whether the gene set enrichment outcomes of subtype-informative markers were preserved in other multi-OMIC cancer data or idiosyncratic in TCGA, we analyzed external datasets of METABRIC [20] (https://www.synapse.org/#!Synapse:syn2133309) and REMBRANDT (GSE6109 [21] and GSE68848 [22]) for breast cancer and GBM respectively. Both datasets consisted of CNV and mRNA expression measurements on the same cohort of patients. We handled mRNA datasets with the same procedures as TCGA data processing, and pre-processed CNV data was directly used in our analysis. The processed METABRIC data contained 16,120 genes with valid entries for both platforms and 1775 samples, while the processed REMBRANDT data contained 6593 genes and 127 samples. 935 and 1143 GO gene sets were selected for METABRIC and REMBRANDT respectively, together with 50 Hallmark gene sets, for MGSEA analysis.

Reproducibility of the gene-level information in external datasets was validated by several methods. First, we sorted genes by their mutual information scores and counted the fractions of overlap between top ranking genes from TCGA and external data with varying rank thresholds (Additional file 10: Figure S4). In general, top ranking genes of both mRNA expression and CNV exhibited reasonable levels of overlap, while mRNA expression achieved higher agreements than CNV. For mRNA expression, about 50% of the top 5000 genes appeared in TCGA and external data for both cancer types. For CNV, the overlap fractions of the top 5000 genes were about 40% for breast cancer and 60% for GBM. The higher overlap fraction in GBM was due to the smaller number of CNV genes in REMBRANDT data. The CNV overlap fraction dropped considerably when the rank threshold decreased, indicating its inferior reproducibility. For instance, in breast cancer data the overlap fraction among the top 2000 genes was about 50% for mRNA expression and 20% for CNV. Second, we solicited several genes undergoing significant subtype-specific copy number alterations according to TCGA references ([2] for breast cancer and [3] for GBM) and checked whether they encountered the same alterations in external data (Additional file 11: Table S7). For breast cancer, HER2-enriched specific amplification of *ERBB2*, basal-specific amplification of *CCNE1*, and luminal B specific amplification of *CCND1* were remarkably salient in both TCGA and METABRIC data. For GBM, proneural specific amplification of *PDGFRA* and classical specific amplification of *EGFR* were strong in TCGA but only moderate in REMBRANDT, while mesenchymal specific deletion of *NF1* was inconspicuous in REMBRANDT data. The weak signal of *NF1* CNV in REMBRANDT was probably due to limited coverage of *NF1* on the platform used by REMBRANDT as previously reported [23]. Third, we plotted the FDR curves of the two external datasets (Additional file 3: Figure S3, the right column) and compared them with their TCGA counterparts. In both datasets, mRNA expression had a much lower FDR than CNV through the entire range of the *p*-value threshold. At the p-value threshold 10^− 10^, the FDRs of mRNA expression were lower than 0.3 in both datasets; the CNV FDR was around 0.8 in METABRIC, while the CNV FDR was close to 1.0 in REMBRANDT.

Additional file 12: Table S8 and Additional file 13: Table S9 report the combinatorial relations of mRNA expression and CNV in 278 functional categories and 143 reduced functional categories for METABRIC breast cancer data. Similar to the TCGA breast cancer data, mRNA expression was a dominant feature in considerably more reduced functional categories than CNV (96 vs 45). However, unlike TCGA the dominant combination of CNV and mRNA expression was only found in 2 reduced functional category. We also counted the identities and numbers of overlapped functional categories for each combinatorial relation between TCGA and METABRIC and report them in Additional file 14: Table S10 and Additional file 15: Table S11. Intriguingly, about half of the reduced functional categories possessing mRNA expression as the only dominant feature appeared in both datasets (161, 199 and 90 reduced functional categories in TCGA, METABRIC and both), as well as for CNV (129, 77 and 36 reduced functional categories in TCGA, METABRIC and both).

Additional file 16: Table S12 and Additional file 17: Table S13 report the combinatorial relations of mRNA expression and CNV in 381 functional categories and 161 reduced functional categories for REMBRANDT GBM data. Similar to TCGA GBM data, the vast majority of reduced functional categories possessed mRNA expression as a dominant feature (341), whereas only a small fraction of reduced functional categories possessed CNV as a dominant feature (39). We also counted the identities and numbers of overlapped functional categories for each combinatorial relation between TCGA and REMBRANDT and report them in Additional file 18: Table S14 and Additional file 19: Table S15. The extent of overlap for TCGA-REMBRANDT comparison was weaker than TCGA-METABRIC comparison but exhibited the same trend. About 60% of the reduced functional categories possessing mRNA expression as the only dominant feature appeared in both datasets (478, 341 and 202 functional categories in TCGA, REMBRANDT and both), whereas the overlap sizes for CNV was about a quarter (77, 39 and 11 functional categories in TCGA, REMBRANDT and both).

We also examined whether the cancer-related processes appeared in each combinatorial enrichment pattern of the two TCGA datasets were retained in external datasets. Intriguingly, most aforementioned observations about the processes in mRNA-dominant and CNV-dominant combinatorial patterns sustained in the external datasets. In METABRIC, mRNA expression was the sole dominant feature in reduced functional categories involved in cell proliferation (such as cell cycle control, DNA replication, estrogen response, *MYC* targets, and *E2F* targets), and CNV was the sole dominant feature in reduced functional categories involved in cell invasion and metastasis (e.g., cell adhesion, EMT) (Additional file 14: Table S10). In REMBRANDT, mRNA expression was the only dominant feature in diverse functional categories such as cell adhesion, angiogenesis, EMT, and inflammatory response (Additional file 18: Table S14). In contrast, only a few reduced functional categories possessed CNV as the only dominant feature in both TCGA GBM and REMBRANDT data. Combinatorial patterns with multiple dominant features had no common functional categories between TCGA and external data.

### Comparison with other GSEA extensions

As alluded in Background, there are numerous extensions of canonical GSEA, yet to our knowledge none of them explicitly intends to capture the combinatorial relations between the enrichment information from multiple platforms. Therefore, directly comparing the performance of MGSEA with other methods is not quite meaningful. Instead we compared MGSEA with six other GSEA extensions in two aspects. First, we contrasted qualitative characteristics of those methods and discussed the pros and cons as well as the adequate utility for each one. Second, we applied one of those methods, moGSA, to the TCGA breast cancer and GBM data and compared their outputs with the inference results of MGSEA.

We chose 6 GSEA extension methods for comparison: moGSA [10], MONA [9], GeneTrail2 [8], Klebanov *et. al.* [6], SetRank [4], and PAGE [5]. Table 3 summarizes the qualitative characteristics of MGSEA and the comparison methods. SetRank, PAGE, and Klebanov et al. address different statistical and computational limitations of the canonical GSEA, and introduce novel algorithms to reduce false positives, to decrease computation time along with increased statistical sensitivity, and to improve multivariate significance testing through multivariate statistics, respectively. The input of these algorithms is identical to that of the canonical GSEA, namely the high-throughput data from one platform (most likely transcriptomic measurements). GeneTrail2 consists of a collection of useful tools to handle GSEA of genomic, proteomic, miRNomics, and transcriptomic data separately. The enrichment outcomes are reported separately and not integrated. Therefore, all those four methods are unimodal in nature.

**Table 3:** A comparison of MGSEA with other GSEA extensions

Algorithm	MGSEA	moGSA	MONA	GeneTrail2	Klebanov *et. al.*	SetRank	PAGE
Data modality	Multimodal	Multimodal	Multimodal	Unimodal	Unimodal	Unimodal	Unimodal
Input	List of sorted genes based on mutual information scores for each feature (CNV, methylation, or mRNA)	Different measurements of gene activity (CNV, mRNA, protein)	Lists indicating whether genes are differentially expressed based on distinct measurement methods (mRNA, protein, microRNA)	A GSE file with data from both sample and reference group, or two GDS files for sample and reference group, or a list of genes and their scores	Single measurement of gene activity	Single measurement of gene activity	Single measurement of gene activity
Output	Graphical display of conditional random walk and its statistical significance	List of pathways and its statistical significance, and inferred activity in individual samples	List of pathways and its statistical significance	List of pathways and its statistical significance	List of pathways and its statistical significance	List of pathways and its statistical significance	List of pathways and its statistical significance
Main Features	Evaluates the combinatorial enrichment relation between biological features	Integration of multiple measurements of a gene into a single score for gene set enrichment analysis	Regulatory relations between features (such as microRNA and its targets) are considered	Each feature (gene, protein, microRNA, and SNP) is tested independently for pathway enrichment	Multivariate N-statistic for multivariate significance testing	Reduces false positives by discarding gene sets identified as significant due to high overlap with another significant gene set	Parametric analysis of GSEA which is less computational intensive and more statistically sensitive

In contrast, both MONA and moGSA incorporate multimodal OMIC measurements. MONA incorporates the regulatory relations between mRNA, microRNA expressions and protein levels in a Bayesian inference algorithm for GSEA. moGSA utilizes multivariable latent variable decomposition to determine the most informative features in each OMIC measurements and calculates an integrated score for each gene set.

Each method has merits and shortcomings as well as adequate utility for its application. SetRank is preferred in reducing the false positives of the resulting gene set, with possible issues regarding sensitivity. PAGE is preferred for increased statistical sensitivity and reduced computational intensity, but possible gene-dependency due to co-regulation of genes might violate the assumptions of the statistical model (normal distribution) used by PAGE. The method by Klebanov et al. is useful when correlation between multiple genes is concerned, but the method is less accessible due to lack of readily available programs/packages. GeneTrail2 offers convenient GSEA analysis for different types of data, but an integrated interpretation of the results from different platforms is not readily available. The strength of MONA lies in its ability to integrate pre-existing knowledge of regulatory relations between features on GSEA, but given the widespread context dependency of such relations [24], outputs based on computationally predicted regulatory relations (such as microRNA and its predicted targets) need to be more cautiously interpreted. moGSA is particularly suited for integrated assessment of GSEA using multiplatform data. However, since only the most informative measurements were incorporated, the relative strength of each feature is not evident in its output. MGSEA explicitly reports the combinatorial relations of enrichment information from multiple platforms, yet it does not generate an aggregate outcome by synthesizing the enrichments from multiple platforms. In this regard, moGSA and MGSEA serve complementary functions.

In order to further elucidate the difference between MGSEA and other methods, we compared the outputs of MGSEA and moGSA by applying both to analyze the breast cancer and GBM TCGA data. The other multimodal method, MONA, was not chosen as it required protein and microRNA expression data, which were beyond the scope of the current analysis. moGSA was run with default parameters, and the outputs were sorted gene sets in terms of their enrichment significance. We varied the threshold of choosing the top-ranking gene sets from moGSA and reported the counts of overlapped gene sets with MGSEA in Table 4. A substantial portion of the functional categories selected by MGSEA were also detected by moGSA (78 and 166 gene sets in breast cancer and GBM data when the top 20% gene sets from moGSA were chosen). Furthermore, MGSEA provides annotations of the combinatorial relations between features in the selected gene sets. These annotations are unique in MGSEA and cannot be generated by moGSA.

**Table 4:** Comparison of MGSEA with moGSA. Overlap of significant gene sets at various cutoffs between moGSA and MGSEA as shown for (A) breast cancer and (B) GBM

Cutoff (%)	Number of gene sets in moGSA	Overlap with MGSEA
A. Breast cancer		
5	56	9
10	112	34
15	168	51
20	225	78
25	281	105
30	337	130
35	393	150
40	449	174
45	505	197
50	562	221
55	618	244
60	674	266
65	730	285
70	786	313
75	842	335
80	898	354
85	955	370
90	1011	393
95	1067	409
100	1123	434
B. GBM		
5	57	44
10	115	80
15	172	124
20	230	166
25	287	207
30	345	240
35	402	277
40	460	308
45	517	338
50	575	367
55	632	398
60	689	424
65	747	452
70	804	481
75	862	517
80	919	550
85	977	577
90	1034	614
95	1092	649
100	1149	676

## Discussion

In this study, we propose MGSEA, an extension of the gene set enrichment analysis to the data from multiple platforms. MGSEA can unravel the combinatorial relations of enrichment information from multiple platforms. For a given gene set, we can tell whether the phenotype-delineating information of a feature is indispensable or subsumed to other features by comparing the random walks of joint features and the corresponding conditional random walks.

MGSEA successfully captured designed feature relations from simulated data. We further investigated a problem of delineating cancer subtypes with biomarkers extracted from multi-OMIC data, and applied MGSEA to TCGA breast cancer and GBM data to identify the combinatorial relations of gene set enrichment information from multiple platforms. The major combinatorial patterns and enriched functional categories in both cancer types possessed both common properties and unique characteristics. In both cancer types, mRNA expression appeared more frequently as a dominant feature than CNV or DNA methylation. In breast cancer, the number of enriched functional categories possessing mRNA expression as a dominant feature (179) moderately surpassed those for CNV (147) and DNA methylation (137). In GBM, the contrast of those numbers for mRNA expression (493), CNV (90) and DNA methylation (150) was much more salient. Furthermore, the enriched functional categories belonging to distinct combinatorial patterns were quite different. In breast cancer, mRNA expression was the only dominant feature in functional categories primarily involved in cell proliferation, such as cell cycle control, estrogen response, DNA repair, *MYC* targets, and *E2F* targets, while CNV was the only dominant feature in functional categories primarily involved in invasion and metastasis, such as cell adhesion and EMT. In GBM, mRNA expression was the only dominant feature in diverse functional categories such as cell adhesion, inflammatory response, angiogenesis, and EMT. In contrast, the combinatorial patterns with multiple dominant or redundant features were much less abundant among enriched functional categories and seemed not concentrated on certain cancer-related processes.

These findings were validated in two independent datasets: METABRIC for breast cancer and REMBRANDT for GBM. For both cancer types (breast cancer and GBM) and the two features (mRNA expression and CNV), about half of the top 5000 genes in terms of mutual information scores occurred in both TCGA and external datasets. The combinatorial patterns of (mRNA expression alone dominant) and (CNV alone dominant) were over-represented in METABRIC, and the combinatorial pattern of (mRNA expression alone dominant) was over-represented in REMBRANDT. The functional categories belonging to those combinatorial patterns were involved in the cancer-related processes analogous to the TCGA data: cell proliferation for mRNA expression and invasion/metastasis for CNV in breast cancer, and diverse processes for mRNA expression in GBM. Furthermore, the functional categories belonging to those combinatorial patterns were largely overlapped between the inferred outcomes of TCGA and external datasets (about 40–60%).

The inferred and validated combinatorial patterns were overwhelmingly concentrated in two scenarios: mRNA expression alone is a dominant feature (for both cancer types), and CNV alone is a dominant feature (for breast cancer). Lack of combinatorial patterns with multiple dominant features implies that in the same functional category informative markers of multiple platforms rarely cover disjoint subsets of members. Rather, informative markers of distinct platforms seem to be enriched in different collections of functional categories.

The abundance of mRNA expression dominant combinatorial patterns and their enriched functional categories are supported by prior studies of cancer subtype classification. Breast cancer and GBM subtypes were originally inferred from mRNA expression data alone (PAM50 genes for breast cancer [25] and a selected panel of 840 genes for GBM [15]). Furthermore, the consensus functional categories of the mRNA expression dominant combinatorial patterns between TCGA and external datasets reflect the underlying biological processes differentiating cancer subtypes, such as cell cycle/proliferation activities [26] and sex hormone activities [27] in breast cancer, and lymphocyte infiltration levels [28] and EMT [29] in GBM. In contrast, the prominence of CNV in subtype delineation and functional enrichment is more striking. CNV and DNA methylation are known to exhibit subtype-specific variations and hence are informative about subtype delineation (such as subtype-specific CNVs reported in TCGA breast cancer [2] and GBM [3] papers, and the overlap of massive CpG island DNA methylation and proneural phenotypes in GBM [30]). However, enrichment of breast cancer CNV biomarkers in cell invasion and metastasis is unexpected. In breast cancer, mRNA expression and CNV seem to alter the two complementary oncogenic processes respectively – cell proliferation and invasion/metastasis – and jointly determine the cancer subtypes.

The FDRs of CNV were generally higher than those of mRNA expression, and were close to 1.0 in REMBRANDT data (Additional file 3: Figure S3). High FDRs of CNV are likely attributed to two causes. First, a chromosomal segment undergoing copy number alteration typically harbors a few driver genes and many more passenger genes. The functional enrichment of the driver genes was therefore considerably diluted. Second, the CNV-subtype associations were much less reproducible than the mRNA expression-subtype associations in external datasets (Additional file 10: Figure S4). In particular, subtype-specific copy number alterations of several well-known driver genes in TCGA GBM were weakly or not reproducible in REMBRANDT data (Additional file 11: Table S7). Despite the poor FDRs, there was still a non-negligible level of overlap between the CNV dominant combinatorial patterns from TCGA and external datasets (about 50% for breast cancer and 25% for GBM). Some of the consensus functional categories likely capture the real associations.

There are many integration algorithms for multi-OMIC data and numerous extensions of the canonical GSEA. However, relatively few methods extend GSEA from a multi-platform data integration perspective, and none of them attempts to capture the combinatorial relations of enrichment information from multiple platforms. MGSEA complements with other integrative GSEA extension algorithms and provides unique annotations (combinatorial relations of multiple platforms) to the enriched gene sets.

The combinatorial patterns inferred from MGSEA specify relations of informative biomarkers in a functional category between multiple platforms. Intuitively, a feature is dominant if its informative biomarkers are not largely contained in informative biomarkers of other features, and two features are redundant if their informative biomarkers are largely overlapped. These simple relations give rise to an exponential number of possible combinatorial patterns when the data of multiple platforms are provided. Among those combinatorial patterns only one of them requires indispensable importance of all platforms – that all features are significantly enriched and dominant. Although this special case clearly demonstrates the benefit of combining all platforms for gene set enrichment, it is only one of many possible combinatorial patterns that can happen. The purpose of this study is to develop a quantitative tool that identifies the combinatorial pattern(s) best supported by the empirical data, rather than verifying a particular hypothesis regarding combinatorial patterns such as the case where all features are dominant. The special case of dominance of all features is not prominent in the cancer multi-OMIC data in this study. Nevertheless, MGSEA identifies several other combinatorial patterns which have important functional implications. For instance, although most functional categories are dominated by single features in breast cancer data, mRNA expression dominates in the functional categories involved in cell proliferation, while CNV dominates in the functional categories involved in invasion and metastasis. Furthermore, without restricting to particular functional categories, the three platforms still provide indispensable information about cancer subtype classification, as the distributions of mutual information scores from the three platforms are generally independent (Additional file 2: Figure S2).

## Conclusions

MGSEA addresses the challenge of integrating multimodal OMIC data and delineating their combinatorial relations. We showed that MGSEA was able to recapitulate known properties regarding cancer subtypes when applied to TCGA breast cancer and GBM data, as well as external METABRIC and REMBRANDT datasets.

However, utility of MGSEA is not restricted to cancer subtype classification. It can be applied to any genotype-phenotype association problem with multimodal genotype data. The same framework can also be generalized to a reciprocal problem where there are multiple phenotype labels and one OMIC feature (such as PheWAS), or an even broader problem where there are multiple phenotype labels and OMIC features. Extension in these directions can help organizing complicated information extracted from those multiple-high dimensional problems.

## Additional files

Additional file 1: **Figure S1.** Bivariate GSEA plot when (A) F1 is superior than F2 (B) F1 and F2 both provide indispensable enrichment information (C) F1 and F2 are largely overlapped in gene set enrichment. The top panels show the locations of genes from a gene set *S* within the sorted list of F1 and F2 genes (identically colored vertical stripes correspond to same genes). (PDF 132 kb)

Additional file 2: **Figure S2.** Correlation of mutual information scores across platforms. Top and bottom panels show the distributions of mutual information scores across platforms for breast cancer and GBM, respectively. For a panel named in the format of “Platform 1 – Platform 2” (e.g. “CNV-MRNA”), top *n*% of the genes in Platform 1 were selected and the corresponding distribution of mutual information scores for these genes in Platform 2 was presented. We varied the percentage threshold of selecting the top-ranking genes and annotated their distributions with distinct colors. (PDF 316 kb)

Additional file 3: **Figure S3.** Relation between FDR and *p*-value cutoff. Each panel shows the relation between FDR and p-value cutoff, with the left column for TCGA data, and the right column for external datasets. (PDF 108 kb)

Additional file 4: **Table S1.** Number of significantly enriched gene sets using top 100 genes from each feature score. The table shows the number of significantly enriched gene sets by querying top 100 genes from each feature score. A gene set is significantly enriched if the p-value of the Fisher’s exact test <0.05. (*XLSX* 8 *kb*)

Additional file 5: **Table S2.** Parameters settings for analysis presented in this study. The table shows the parameters used for MGSEA and REVIGO in the analysis. (XLSX 8 kb)

Additional file 6: **Table S3.** Mann-Whitney *p*-values of 16 pairwise random walk comparisons among the 434 functional categories in the TCGA breast cancer data. The table shows the Mann-Whitney p-values of 16 pairwise random walk comparisons among the 434 significant gene sets in the TCGA breast cancer data. (XLSX 79 kb)

Additional file 7: **Table S4.** Dominant and combinatorial features of enrichment in 434 functional classes of TCGA breast cancer data. The table shows the full list of significantly enriched gene sets and their combinatorial relations for TCGA breast cancer data. The reduced functional category for each gene set is also reported. An NA entry denotes that the gene set and its reduced functional category are identical. (XLSX 28 kb)

Additional file 8: **Table S5.** Mann-Whitney p-values of 16 pairwise random walk comparisons among the 676 functional categories in the TCGA GBM data. Mann-Whitney p-values of 16 pairwise random walk comparisons among the 676 significant gene sets in the TCGA GBM data. (XLSX 118 kb)

Additional file 9: **Table S6.** Dominant and combinatorial features of enrichment in 676 functional classes of TCGA GBM data. The table shows the full list of significantly enriched gene sets and their combinatorial relations for TCGA GBM data. The reduced functional category for each gene set is also reported. An NA entry denotes that the gene set and its reduced functional category are identical. (XLSX 38 kb)

Additional file 10: **Figure S4.** Overlap between top ranking genes from TCGA and external data. Top panels show the overlap of top ranking genes between TCGA breast cancer and METABRIC, while the bottom panels show the overlap between TCGA GBM data and REMBRANDT. Left and right panels display the overlap for CNV and mRNA for each cancer type, respectively. (PDF 109 kb)

Additional file 11: **Table S7.** Subtype-specific CNV and their values in TCGA and external dataset. The table show the previously reported subtype-specific CNV and their values (mean and standard deviation) for (A) breast cancer and (B) GBM TCGA and external dataset. The values were CDFs (ranged from 0 to 1) for TCGA data, log_2_ of estimated copy numbers (centered at 0) for METABRIC, and estimated copy numbers (centered at 2) for REMBRANDT, respectively. (XLSX 11 kb)

Additional file 12: **Table S8.** Dominant and redundant features of enrichment in 278 functional classes of METABRIC data. The table shows the full list of significantly enriched gene sets and their combinatorial relations for METABRIC data. The reduced functional category for each gene set is also reported. An NA entry denotes that the gene set and its reduced functional category are identical. (XLSX 19 kb)

Additional file 13: **Table S9.** Dominant and redundant features of enrichment in 143 reduced functional classes of METABRIC data. The table shows the reduced list of significantly enriched gene sets and their combinatorial relations for METABRIC data. (XLSX 13 kb)

Additional file 14: **Table S10.** Identities of significant gene set overlap between TCGA breast cancer and METABRIC data. The table shows the full list of significantly enriched gene set overlap between TCGA breast cancer and METABRIC data. (XLSX 13 kb)

Additional file 15: **Table S11.** Number of significant gene set overlap between TCGA breast cancer and METABRIC data and their combinatorial patterns. The table shows the number and combinatorial pattern of significantly enriched gene set overlap between TCGA breast cancer and METABRIC data. (XLSX 9 kb)

Additional file 16: **Table S12.** Dominant and redundant features of enrichment in 381 functional classes of REMBRANDT data. The table shows the full list of significantly enriched gene sets and their combinatorial relations for REMBRANDT data. The reduced functional category for each gene set is also reported. An NA entry denotes that the gene set and its reduced functional category are identical. (XLSX 22 kb)

Additional file 17: **Table S13.** Dominant and redundant features of enrichment in 161 reduced functional classes of REMBRANDT data. The table shows the reduced list of significantly enriched gene sets and their combinatorial relations for REMBRANDT data. (XLSX 12 kb)

Additional file 18: **Table S14.** Identities of significant gene set overlap between TCGA GBM and REMBRANDT data. The table shows the list of significantly enriched gene set overlap between TCGA GBM and REMBRANDT data. (XLSX 15 kb)

Additional file 19: **Table S15.** Number of significant gene set overlap between TCGA GBM and REMBRANDT data and their combinatorial patterns. The table shows the number and combinatorial pattern of significantly enriched gene set overlap between TCGA GBM and REMBRANDT data. (XLSX 11 kb)

Additional file 20: **Supplementary File S1.** The R source codes of the MGSEA program, a toy example dataset, and a brief explanation for running the program. (ZIP 1832 kb)

## Abbreviations

CDF: Cumulative distribution function
CNV: Copy number variation
EMT: Epithelial-mesenchymal transition
FDR: False discovery rate
GBM: Glioblastoma multiforme
GO: Gene ontology
GSEA: Gene set enrichment analysis
METABRIC: Molecular Taxonomy of Breast Cancer International Consortium
MGSEA: Multivariate gene set enrichment analysis
MSigDB: Molecular signatures database
REMBRANDT: Repository for molecular brain neoplasia data
SNP: Single nucleotide polymorphism
TCGA: The Cancer Genome Atlas
