# Supplementary figures and images for: MGSEA – a multivariate Gene set enrichment analysis
Source: BMC Bioinformatics. 2019 Mar 18;20:145. doi: 10.1186/s12859-019-2716-6 (PMC6421703; doi:10.1186/s12859-019-2716-6)

A

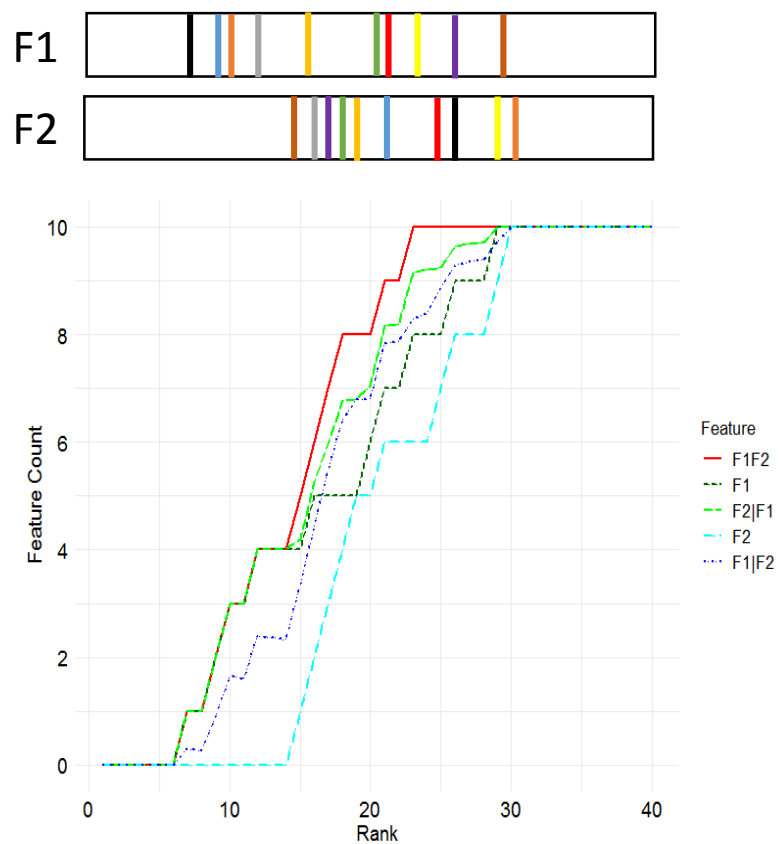

B

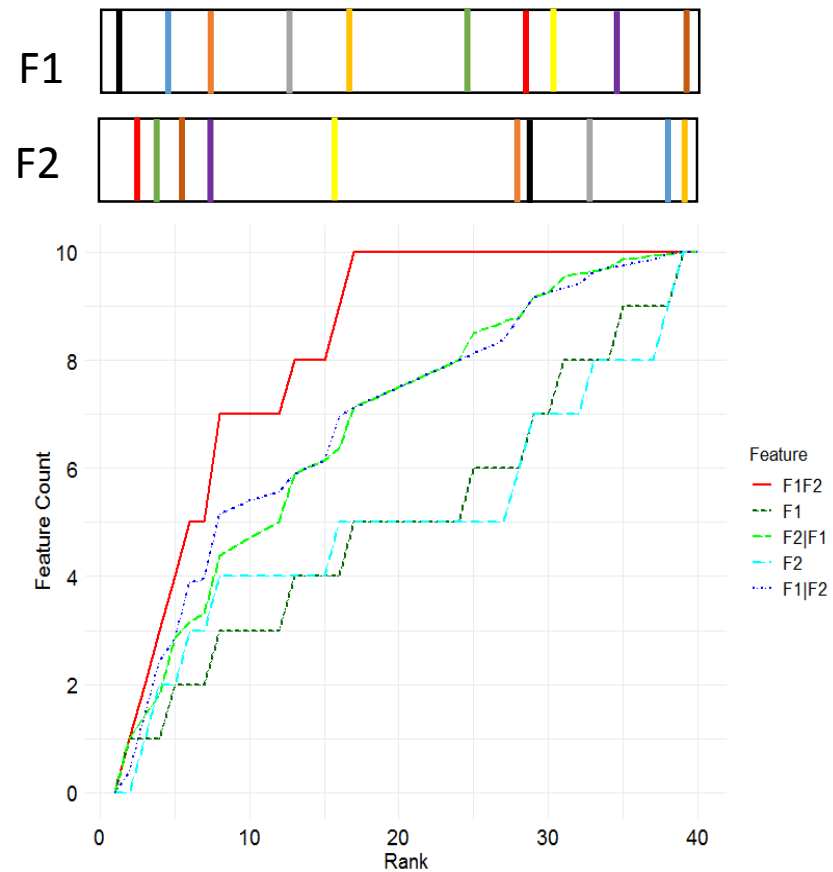

C

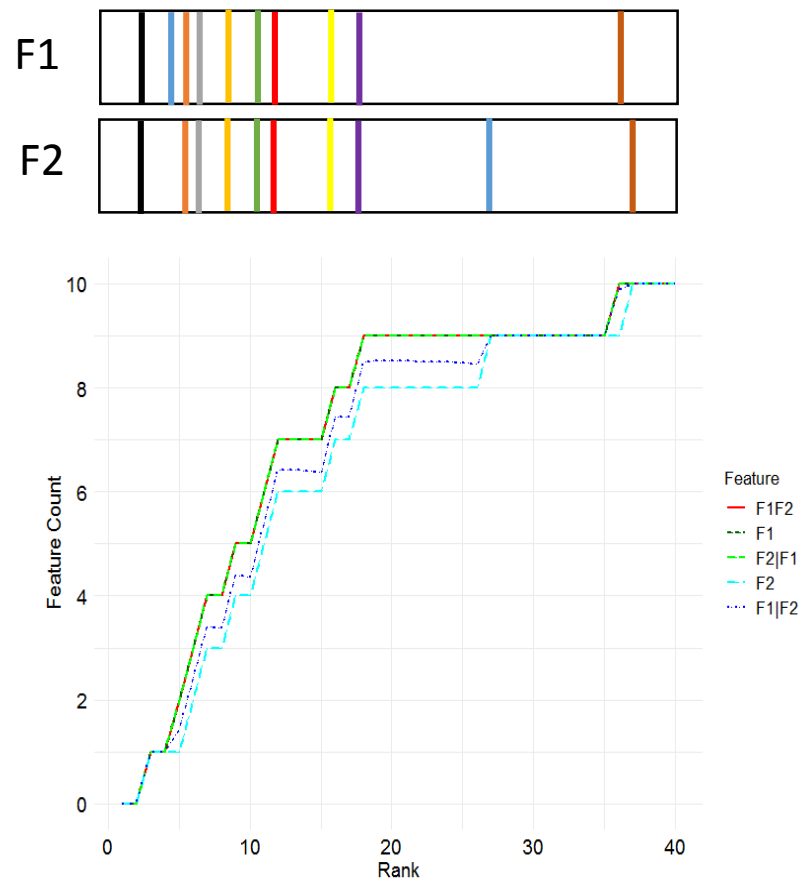

Supplement: Supplementary file 1 — Figure S1. Bivariate GSEA plot when (A) F1 is superior than F2 (B) F1 and F2 both provide indispensable enrichment information (C) F1 and F2 are largely overlapped in gene set enrichment. The top panels show the locations of genes from a gene set S within the sorted list of F1 and F2 genes (identically colored vertical stripes correspond to same genes). (PDF 132 kb) [file 12859_2019_2716_MOESM1_ESM.pdf]

# BRCA

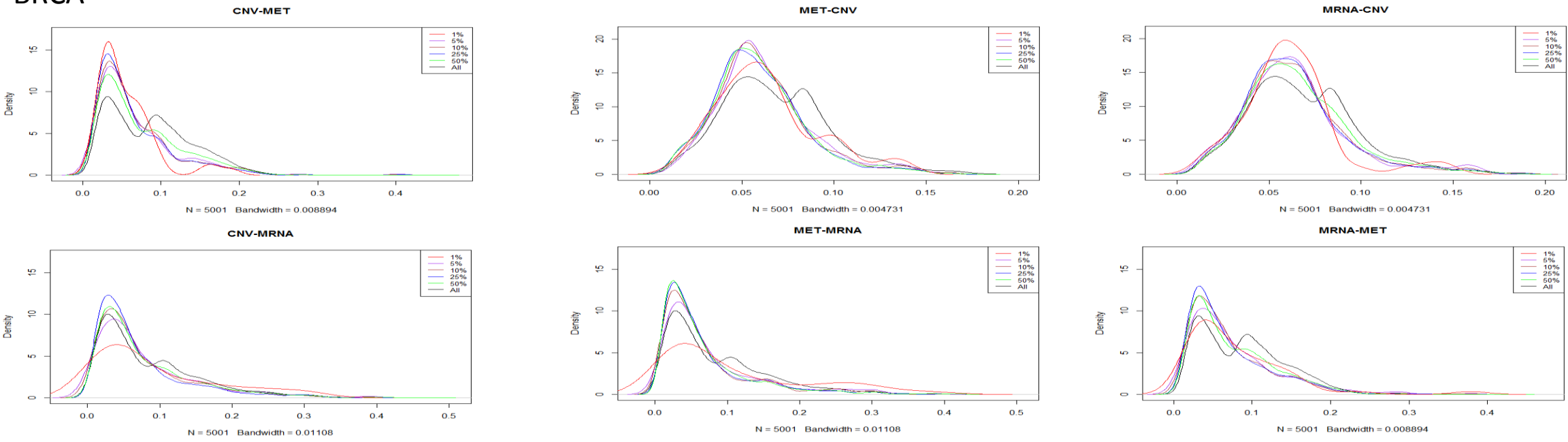

# GBM

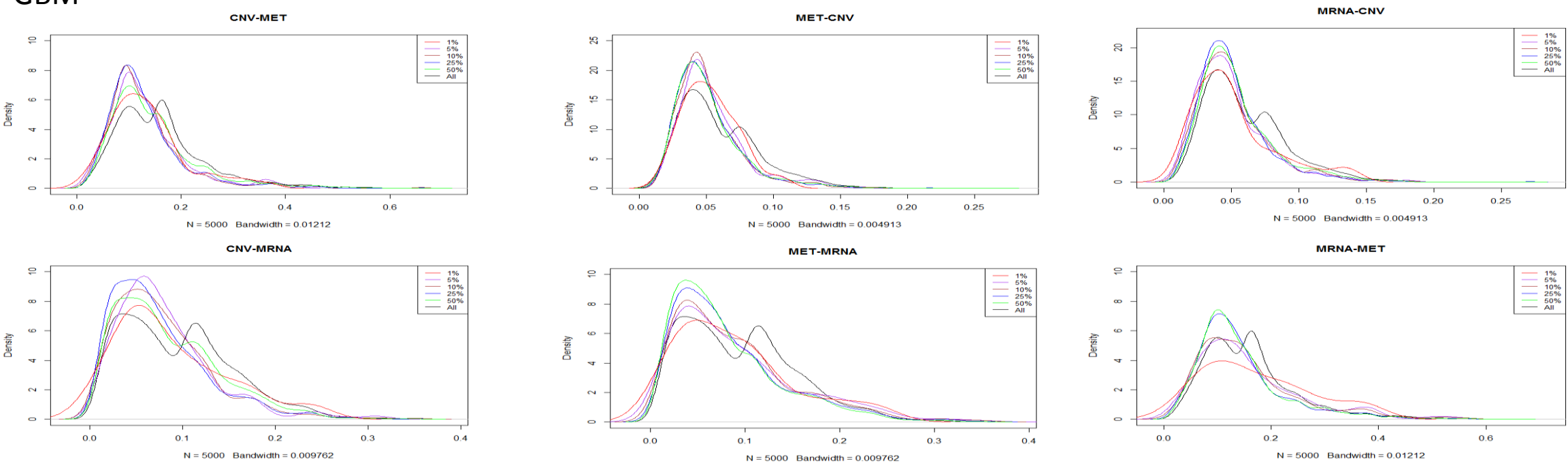

Supplement: Supplementary file 2 — Figure S2. Correlation of mutual information scores across platforms. Top and bottom panels show the distributions of mutual information scores across platforms for breast cancer and GBM, respectively. For a panel named in the format of “Platform 1 – Platform 2” (e.g. “CNV-MRNA”), top n% of the genes in Platform 1 were selected and the corresponding distribution of mutual information scores for these genes in Platform 2 was presented. We varied the percentage threshold of selecting the top-ranking genes and annotated their distributions with distinct colors. (PDF 316 kb) [file 12859_2019_2716_MOESM2_ESM.pdf]

**BRCA**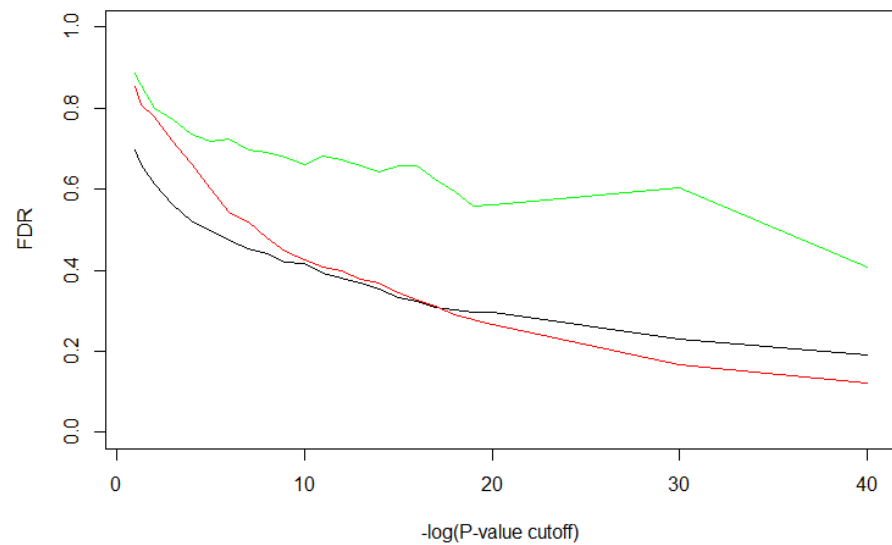**METABRIC**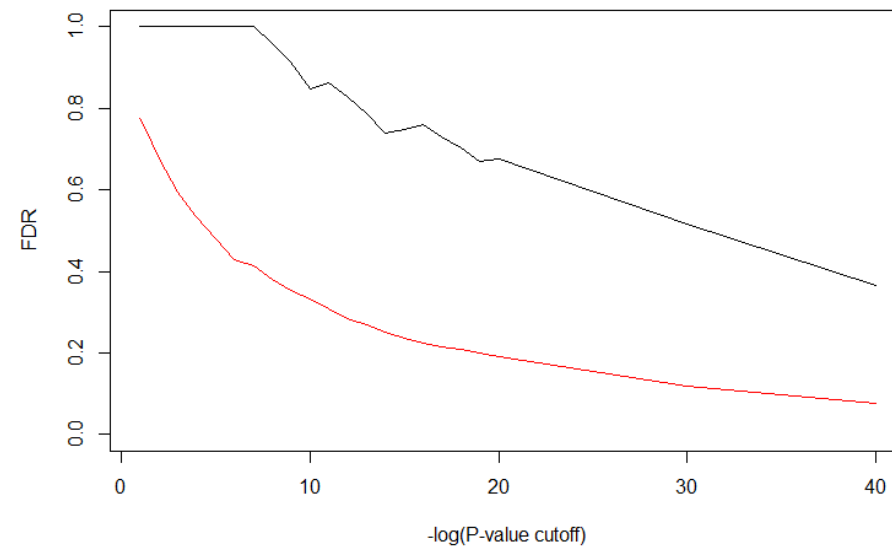**GBM**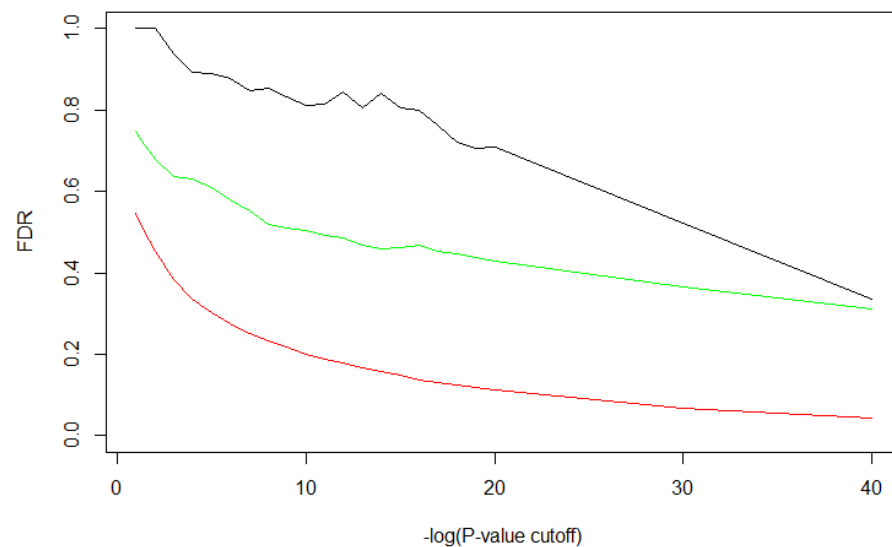**REMBRANDT**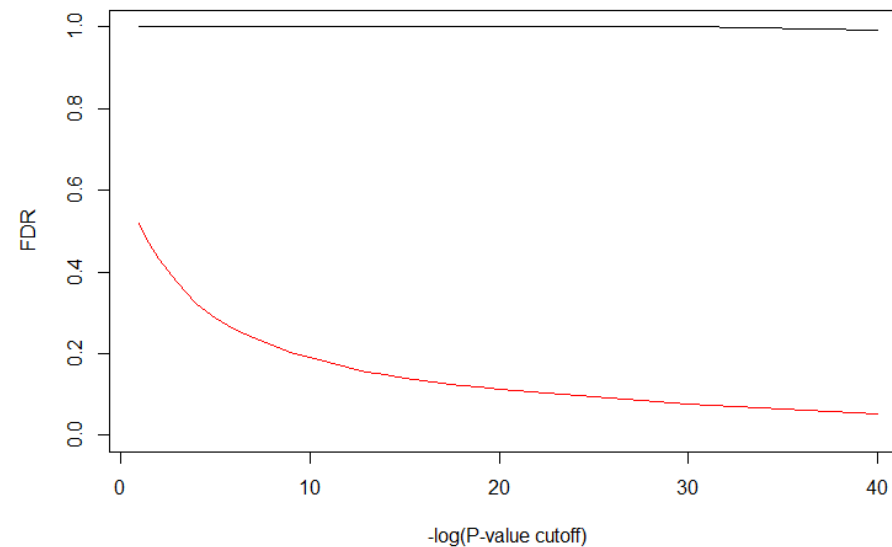**— CNV****— MET****— MRNA**

Supplement: Supplementary file 3 — Figure S3. Relation between FDR and p-value cutoff. Each panel shows the relation between FDR and p-value cutoff, with the left column for TCGA data, and the right column for external datasets. (PDF 108 kb) [file 12859_2019_2716_MOESM3_ESM.pdf]

BRCA

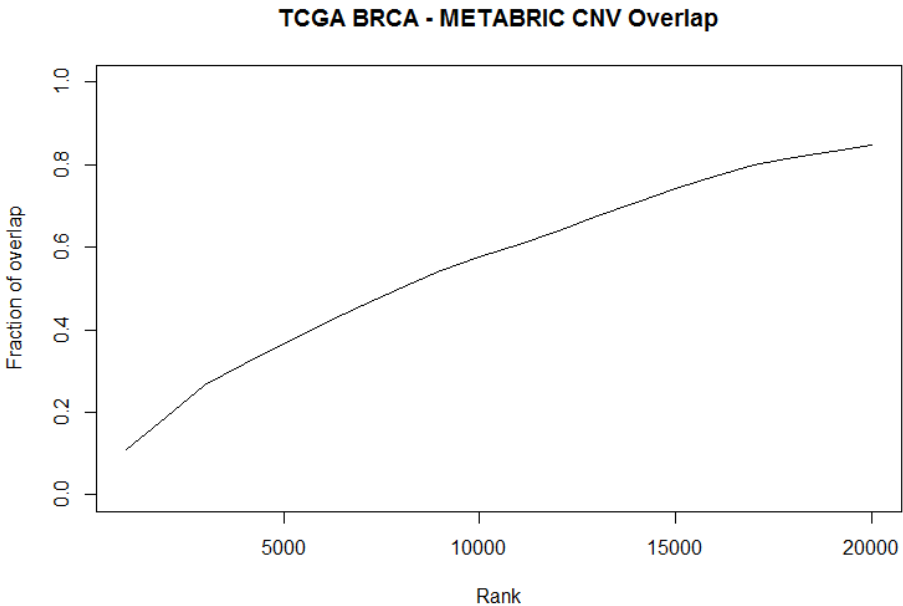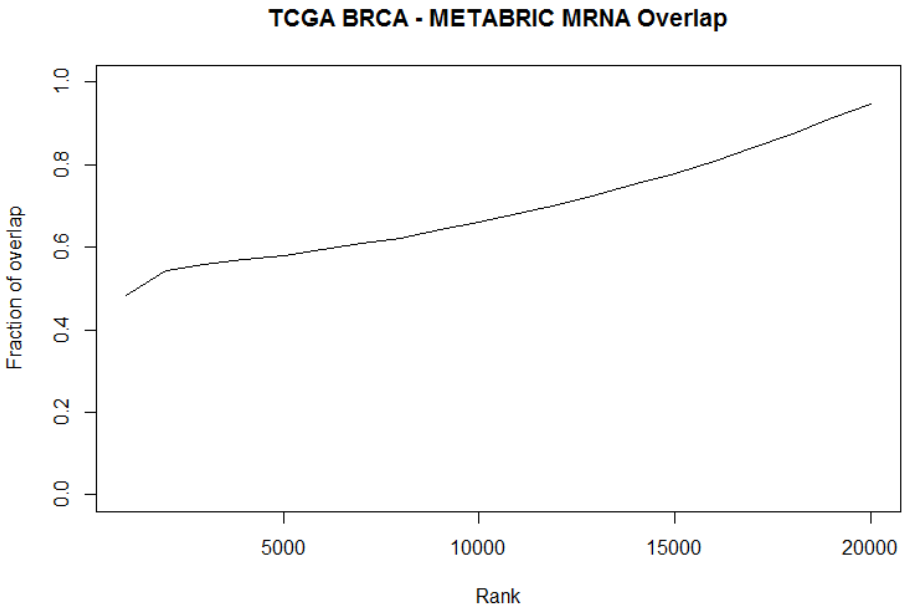

GBM

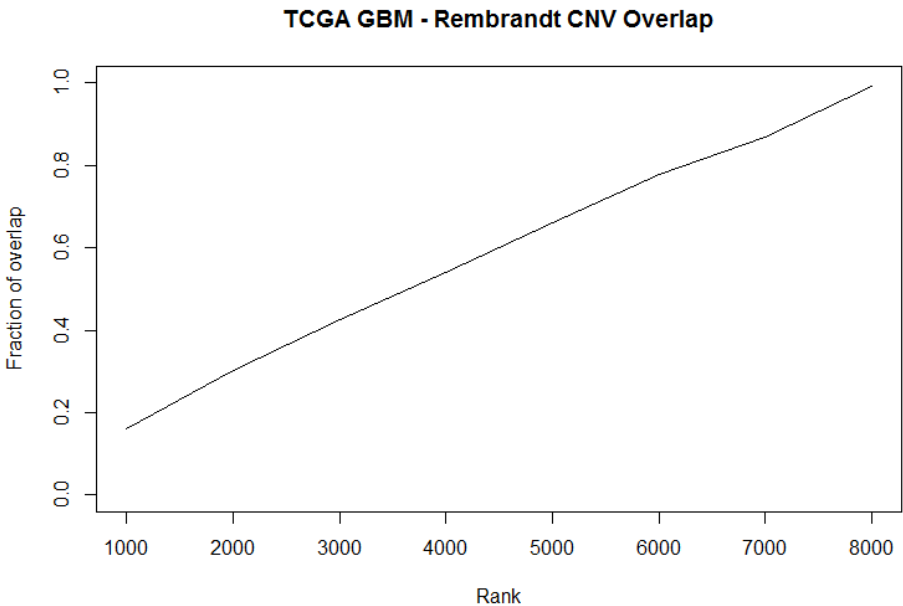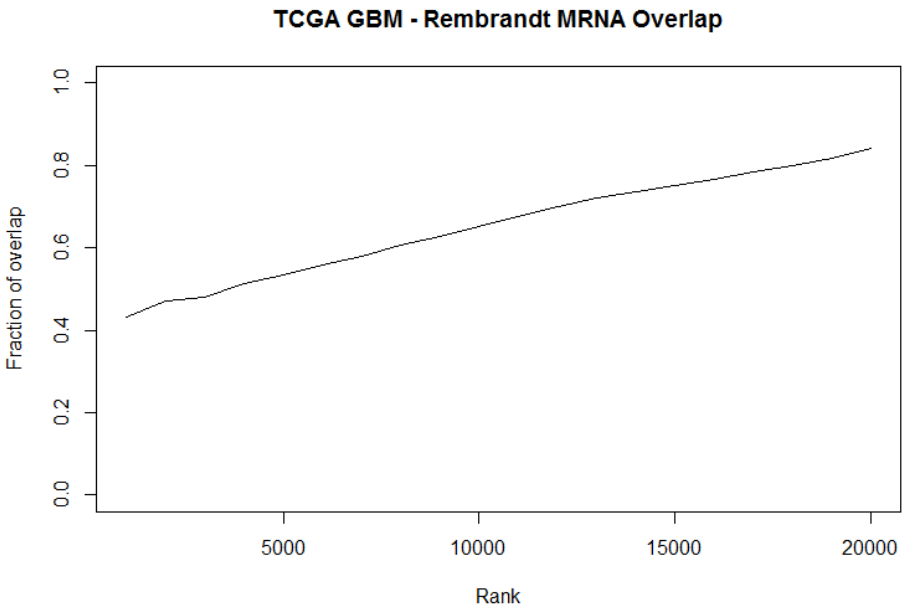

Supplement: Supplementary file 10 — Figure S4. Overlap between top ranking genes from TCGA and external data. Top panels show the overlap of top ranking genes between TCGA breast cancer and METABRIC, while the bottom panels show the overlap between TCGA GBM data and REMBRANDT. Left and right panels display the overlap for CNV and mRNA for each cancer type, respectively. (PDF 109 kb) [file 12859_2019_2716_MOESM10_ESM.pdf]
