# Supplementary material for: MGSEA – a multivariate Gene set enrichment analysis
Source: BMC Bioinformatics. 2019 Mar 18;20:145. doi: 10.1186/s12859-019-2716-6 (PMC6421703; doi:10.1186/s12859-019-2716-6)
Supplement: Supplementary file 20 — Supplementary File S1. The R source codes of the MGSEA program, a toy example dataset, and a brief explanation for running the program. (ZIP 1832 kb) [file 12859_2019_2716_MOESM20_ESM.zip › SampleCode/Readme.pdf]

Instructions for running MGSEA analysis as reported in the manuscript "MGSEA – A Multivariate Gene Set Enrichment Analysis".

Software used:

1. RStudio 1.0.136 with R 3.2.2.

Part I

Required Input Files

1. Mapping between a gene with its Gene Ontology ID (GOID), processed from annotation file "goa_human.gaf" downloaded from Gene Ontology Consortium. A sample is provided as "Gene_Symbol_GOID_mapping.csv"

2. Mapping between GOID and the name of the process, processed from ontology file "go.obo" downloaded from Gene Ontology Consortium. A sample is provided as "GOID_to_Name_Mapping.csv".

3. A list of selected GOID to be analyzed by MGSEA. A sample is provided as "GOID.csv".

4. A list of precomputed values of Log(N!), provided as "LogNFactorial.csv".

5. A list of hallmark gene sets downloaded from Molecular Signatures Database (MSigDB) (http://software.broadinstitute.org/gsea/msigdb).

6. A list of sorted top-scoring genes according to designated scoring criteria (ANOVA or mutual information). A sample is provided as "Top1000Genes.csv", which contains the shared top-1000 scoring genes for the factors CNV, MET and MRNA.

Part II

Running MGSEA analysis

1. To run MGSEA on Gene Ontology gene sets, please use "MGSEAGO.R".

   1.1 Input files are as listed in Part 1, subsections 1, 2, 3, 4, and 6.

   1.2 Output files are individual text files, named after each gene set, with p-values for CNV vs control, MET vs control, MRNA vs control, CNV&MET vs MET|CNV, CNV&MET vs CNV|MET, CNV&MRNA vs MRNA|CNV, CNV&MRNA vs MRNA|CNV, MET&MRNA vs MRNA|MET, MET&MRNA vs MET|MRNA, CNV&MRNA&MET vs MRNA|CNV&MET, CNV&MRNA&MET vs MET|CNV&MRNA, CNV&MRNA&MET vs MRNA|CNV&MET, CNV&MET vs control, MET&MRNA vs control, CNV&MRNA vs control, CNV&MRNA&MET vs control, respectively.

   1.3 Corresponding plots of the aforementioned comparisons, in PDF format will also be generated

2. To run MGSEA on hallmark gene sets, please use "MGSEAHallmark.R".

2.1 Input files are as listed in Part 1, subsections 4,5, and 6.

2.2 Output files are individual text files, named after each gene set, with p-values for CNV vs control, MET vs control, MRNA vs control, CNV&MET vs MET|CNV, CNV&MET vs CNV|MET, CNV&MRNA vs MRNA|CNV, CNV&MRNA vs MRNA|CNV, MET&MRNA vs MRNA|MET, MET&MRNA vs MET|MRNA, CNV&MRNA&MET vs MRNA|CNV&MET, CNV&MRNA&MET vs MET|CNV&MRNA, CNV&MRNA&MET vs MRNA|CNV&MET, CNV&MET vs control, MET&MRNA vs control, CNV&MRNA vs control, CNV&MRNA&MET vs control, respectively.

2.3 Corresponding plots of the aforementioned comparisons, in PDF format will also be generated
